# The microglial innate immune receptor TREM2 participates in fear memory formation through excessive prelimbic cortical synaptic pruning

**DOI:** 10.3389/fimmu.2024.1412699

**Published:** 2024-10-31

**Authors:** Le-le Zhang, Peng Cheng, Yuan-qing Chu, Zi-ming Zhou, Rong Hua, Yong-mei Zhang

**Affiliations:** ^1^ National Medical Products Administration Key Laboratory for Research and Evaluation of Narcotic and Psychotropic Drugs, Xuzhou Medical University, Xuzhou, China; ^2^ Jiangsu Province Key Laboratory of Anesthesiology, Xuzhou Medical University, Xuzhou, China; ^3^ Jiangsu Province Key Laboratory of Anesthesia and Analgesia Application Technology, Xuzhou Medical University, Xuzhou, China; ^4^ Department of Emergency, The Affiliated Hospital of Xuzhou Medical University, Xuzhou, China

**Keywords:** fear memory formation, prelimbic, microglia, synaptic pruning, TREM2

## Abstract

**Introduction:**

Fear memory formation has been implicated in fear- and stress-related psychiatric disorders, including post-traumatic stress disorder (PTSD) and phobias. Synapse deficiency and microglial activation are common among patients with PTSD, and induced in animal models of fear conditioning. Increasing studies now focus on explaining the specific mechanisms between microglia and synapse deficiency. Though newly-identified microglia regulator triggering receptor expressed on myeloid cells 2 (TREM2) plays a role in microglial phagocytic activity, its role in fear-formation remains unknown.

**Methods:**

We successfully constructed a fear- formation model by foot-shock. Four days after foot-shock, microglial capacity of synaptic pruning was investigated via western blotting, immunofluorescence and Golgi-Cox staining. Prelimbic chemical deletion or microglia inhibition was performed to detect the role of microglia in synaptic loss and neuron activity. Finally, Trem2 knockout mice or wild-type mice with Trem2 siRNA injection were exposed to foot-shock to identify the involvement of TREM2 in fear memory formation.

**Results:**

The results herein indicate that the foot-shock protocol in male mice resulted in a fear formation model. Mechanistically, fear conditioning enhanced the microglial capacity for engulfing synapse materials, and led to glutamatergic neuron activation in the prelimbic cortex. Prelimbic chemical deletion or microglia inhibition improved fear memory formation. Further investigation demonstrated that TREM2 regulates microglial phagocytosis, enhancing synaptic pruning. Trem2 knockout mice showed remarkable reductions in prelimbic synaptic pruning and reduced neuron activation, with decreased fear memory formation.

**Discussion:**

Our cumulative results suggest that prelimbic TREM2-mediated excessive microglial synaptic pruning is involved in the fear memory formation process, leading to development of abnormal stress-related behavior.

## 1 Introduction

Fear is a protective emotion during exposure to environmental dangers, and is readily available to ensure that animals respond promptly to similar stimuli to avoid harm (1). However, continued response may become detrimental once the traumatic event has passed, if it surpasses the animal’s allostatic capacity; at this point it can manifest as symptoms of re-experiencing, avoidance, and hyperarousal (2), eventually directly harming health. Thus, fear memory formation is closely associated with fear-related disorders, including post-traumatic stress disorder (PTSD) and phobias (3). Although a growing body of evidence has revealed that immune alterations may be part of PTSD development in humans (4), the specific mechanisms underlying the role of microglia in this process remain unknown. It is thus essential to identify the potential molecular mechanisms involved, to optimize treatment strategies.

Accumulative evidence has implicated the medial prefrontal cortex as being highly contributing to fear memory (5–9). Importantly, this region is subdivided into the prelimbic and infralimbic areas, which are functionally and anatomically distinct in the fear memory process (10). Fear conditioned mouse models have shown that prefrontal somatostatin interneuron activity is an important causal mechanism of fear memory expression (7). Furthermore, while prelimbic inactivation inhibits fear memory expression, it does not alter fear extinction. In contrast, infralimbic inactivation does not impact fear memory expression but does inhibit fear extinction (11). The prelimbic and infralimbic areas also have complex functions in fear expression and extinction, with some studies suggesting that both appear to have similar effects on neural activity patterns under certain conditions (12). This cumulative evidence shows that the prelimbic area is biased towards fear expression and the infralimbic towards fear extinction, and that the underlying mechanisms for this require further study.

Microglia, a major cell population in the innate immune system (13, 14), have a functional housekeeping role, in which they continuously promote tissue homeostasis and monitor synaptic plasticity under both healthy and imbalanced conditions (15). Developmentally, microglia have emerged as major players in synaptic pruning, which they perform in a classic complement-dependent manner (e.g., complements C3 and C1q) (16, 17). Microglia have the capacity to engulf of the pre- and/or post-synapse, dynamically altering contacts among hippocampal and cerebral cortex synaptic elements during the first postnatal weeks (18). In principle, the microglia can also remove less active synapses to prioritize those that are more active in early development (19). It has recently become evident that microglia may exacerbate phagocytic activity to eliminate ‘superfluous’ synaptic connections in certain neuropsychiatric diseases, including Alzheimer’s and Parkinson’s (20, 21). Unsurprisingly, recent insights into microglial function in PTSD pathogenesis indicates that they increase alterations from synaptic pruning (22), and that fear conditioning triggers neuronal dendritic spine loss in the medial prefrontal cortex (23). Thus, targeted study of microglia-mediated synaptic pruning dysregulation may have translational value in the treatment of stress-related disorders, and may lead to significant improvements in therapeutic outcomes.

The triggering receptor expressed on myeloid cells 2 (TREM2) is a single-pass transmembrane receptor of the immunoglobulin superfamily, initially described as selectively expressed in myeloid-derived (e.g., monocytes, neutrophils, macrophages, osteoclasts) and glial cells (24–26). In the central nervous system (CNS), TREM2 is particularly enriched on microglia and its transduction pathways play critical roles in restraining detrimental inflammation and, contributing, among other actions, to promoting microglial phagocytosis (25, 27, 28). Microglial phagocytic activity has also been associated with certain neuropsychiatric disorders (29, 30). Mice has displayed synapse loss, microglial activation, and increased synaptic pruning of activated hippocampal microglial cells microglial cells on days7 and 26 after fear conditioning. Additionally, probiotic treatment has been shown to rescue this effect, through mitigation of synaptic pruning of activated microglial cells (22). After electric foot-shock exposure in a contextual fear memory study, the level of postsynaptic proteins, including discs large MAGUK scaffold protein 4 (Dlg4, the encoding gene for postsynaptic density protein 95 [PSD95]), SH3, and multiple ankyrin repeat domains 1 (Shank1), Shank2, and Shank3 were all remarkably decreased in hippocampus (31).Among the most significant potential modulators of microglial phagocytic activity, TREM2 is bound in its disease role to nanomolar concentrations of ApoE and aminophospholipids like phosphatidylserine (32, 33). These advances suggest that TREM2-dependent engulfment via microglia may represent a novel therapeutic fear memory target.

Herein, we used paired conditioned stimulus (CS) and unconditioned stimulus (US) to establish a mouse fear conditioning model, to investigate microglial involvement in the fear memory formation process via TREM2-dependent synapse engulfment patterns. We identified that both microglial engulfment capacity and TREM2 expression are increased in the prelimbic area, and that this leads to excessive synaptic pruning and, ultimately, synapse loss. These cumulative findings suggest that targeting TREM2 may lead to advances in developing precision medicine for patients with fear-related disorders and may improve their quality of life.

## 2 Materials and methods

### 2.1 Animals

Male wild-type (WT) C57BL/6J mice (7–9 weeks old, 22–25 g) and Trem2 knockout (KO) (B6/JGpt-Trem2em1Cd3332in1/Gpt) mice on the C57BL/6 background were procured from Gempharmatech Co., Ltd (Nanjing, China). The total number of male WT and Trem2 KO mice were 355 and 58, respectively. Animals were maintained at 22 ± 2°C and 50 ± 10% humidity, under a standard 12-h light/dark cycle, and group-housed (3–5 mice in each cage). Mice had ad libitum access to standard rodent chow and water. Experiments and analyses were performed double-blinded. All experiments were conducted in accordance with the National Institutes of Health Guide for the Care and Use of Laboratory Animals and approved by the Institutional Animal Care and Use Committee at Xuzhou Medical University.

### 2.2 Fear conditioning and testing

The fear conditioning paradigm was adapted from methods previously described by Zhang et al. (34). Briefly, the entire procedure took three days in two contexts. On the first day, mice were placed in the behavioral laboratory for 30 min to ensure adaptation to the novel environment. Then, they were allowed to freely explore Context A (black polyester fiber panels, stainless-steel grid floor) during a 10-min context pre-exposure session. The following day, after the 30-min behavioral laboratory acclimatization, mice were randomly divided into groups and placed in Context A where they were either exposed to three pairings of the CS (a sound, 30 s, 90 dB, 8,000 Hz) and US (foot-shock, 1 s, 1 mA) or (for the control group) exposed to only the CS (a sound, 30 s, 90 dB, 8,000 Hz) three times. During the pairings, mice were allowed to explore their surroundings for 60 s to record baseline freezing levels, followed by three 30-s tones, each of which was delivered at with an interval of 210 s and co-terminated with a 1-s foot-shock. After 4 days, mice were being reintroduced to Context A, and remained there without sound stimulation or foot-shock for a 5-min contextual test to record freezing levels. At least 2 h later, they were placed into Context B (white polyester fiber panels, white acrylic solid floor) for the cued fear test, during which they experienced a 60-s exploration period and a 30-s sound CS. Freezing levels were recorded with a near-infrared FireWire video camera (Med Associates, St. Albans, VT) attached to a computer.

### 2.3 Stereotactic injections

For chemogenetic inhibition (activation), viral vectors and viral vector plasmids were obtained from BrainVTA (Wuhan, China). Mice were anesthetized with 1% sodium pentobarbital (60 ml/kg, intraperitoneal [i.p.]) or with 2–5% isoflurane (R500, RWD Life Science, China), then immobilized on a stereotaxic device (68046, RWD Life Science). Next, a midline incision and two small holes were made in the skull. We then injected 300 nl of rAAV-CaMKIIα-hM3D(Gq)-mCherry-WPRE-hGH-pA, rAAV-CaMKIIα-hM4D(Gi)-mCherry-WPRE-hGH-pA or rAAV-CaMKIIα-mCherry-WPRE-hGH-pA which payload are AAV 2/9 into the bilateral prelimbic (AP, +1.98 mm; ML, ± 0.30 mm; DV, −2.20 mm; relative to bregma) at 0.07 µL/min via a microinjection pump. Post-injection, we waited 10 min to ensure full solution diffusion, then closed and sterilized the wound. All behavior tests were conducted at least 3 weeks post-surgery. After all behavior tests were completed, immunofluorescence staining was performed to verify virus localization accuracy, as follows: male mice were deeply anesthetized, after which they were administered serial intracardial perfusions with 0.9% cold saline (100 mL/100 g), and 4% paraformaldehyde (PFA; 100 mL/100 g). Whole brains were rapidly removed and postfixed in 4% PFA for 24 h at 4°C and cryoprotected in 30% sucrose at 4°C. Sections (30 μm thick) were prepared with a Leica freezing microtome. After they were washed in PBS three times, brain sections were mounted on slides and counterstained with DAPI staining solution. Images were captured using confocal microscopy (Olympus, Tokyo, Japan). All bacterial and viral strains used herein are listed in **Table 1**. To study the effects of minocycline (10 µg/µL, 0.1 µL/site, Sigma, USA) and Trem2 siRNA (Generay, China: 5′-GGACCCUCUAGAUGACCAATT-3′ and 3′-UUGGUCAUCUAGAGGGUCCTT-5′) microinjections on fear formation, we injected these into the bilateral prelimbic area under anesthesia and examined behavioral performance post-administration.

**Table 1:** Experimental bacterial and virus strains used in this article.

Bacterial and virus strains	Manufacturer, Catalog Number
rAAV-CaMKIIα-hM4D(Gi)-mCherry-WPRE-hGH-pA, AAV 2/9	BrainVTA, PT-0017
rAAV-CaMKIIα-hM3D(Gq)-mCherry-WPRE-hGH-pA, AAV 2/9	BrainVTA, PT-0049
rAAV-CaMKIIα-mCherry-WPRE-hGH-pA, AAV 2/9	BrainVTA, PT-0108

### 2.4 Chemogenetic neuronal manipulation

Glutamatergic neurons were labeled by AVV-CaMKIIα-hM4D(Gi)-mCherry-WPRE-pA or AVV-CaMKIIα-hM3D(Gq)-mCherry-WPRE-pA to inhibit or activate prelimbic glutamatergic neurons. Twenty-one days after microinjection, hM4D(Gi)/hM3D(Gq) was activated by the selective ligand clozapine N-oxide (CNO, i.p. HY-17366, MCE, USA) dissolved in 2% DMSO and then diluted with sterile saline. Mice were administered CNO at a final injection volume of 0.3 mL/kg 1 h before fear response assessment, as previously reported before (35).

### 2.5 Western blotting

After they were deeply anesthetized with isoflurane, mice were quickly decapitated and their brain tissues containing the bilateral object regions were isolated with liquid nitrogen, then placed in an Eppendorf tube and stored at -80°C until needed. Total proteins of the prelimbic subregion tissue were lysed in RIPA lysis buffer (500 μL/200 mg) (P00138, Beyotime, China) with phosphatase inhibitor phenylmethylsulfonyl fluoride (1 mM) (ST506, Beyotime, China). Protein concentrations were detected and trimmed using the BCA protein assay kit (P0012, Beyotime, China). Equal amounts of proteins were loaded per lane, separated by SDS-PAGE gels (P0015, Beyotime, China) and transferred to PVDF membranes. The blot was blocked with 5% non-fat milk at room temperature (RT) for 2 h, followed by incubating with primary antibodies TREM2, postsynaptic density protein 95 (PSD95), synaptophysin (SYP), tubulin beta or β-actin overnight at 4°C. After washing with TRIS-buffered saline with Tween, the PVDF membranes were incubated with an HRP-conjugated secondary antibody at RT for 1 h. Protein bands were visualized using the BeyoECL Moon kit (P0018, Beyotime, China), then analyzed with ImageJ software (NIH, USA). All primary and secondary antibodies used herein are listed in **Table 2**.

**Table 2:** Experimental antibodies used in this article.

Antibodies	Host	Manufacturer, Catalog Number	Application	Working dilutions or concentrations
TREM-2	Sheep	R&D Systems, AF1729	WB	1:500
PSD95	Rabbit	Invitrogen, 51-6900	WB, IF	1:500
Synaptophysin	Rabbit	Proteintech, 117785-1-AP	WB, IF	1:500
β-actin	Rabbit	Proteintech, 66009-1-Ig	WB	1:2000
Tubulin	Rabbit	Affinity, DF7967	WB	1:3000
Anti-rabbit IgG, HRP	Goat	Proteintech, SA00001-2	WB	1:2000
Anti-sheep IgG, HRP	Rabbit	Proteintech, SA00001-16	WB	1:2000
Iba1	Mouse	Abcam, ab283319	IF	1:400
Iba1	Rabbit	Cell signaling, 17198S	IF	1:400
CD68	Rat	BioRad, MCA1957T	IF	1:400
CaMKII	Mouse	Thermo Fisher, MA1-048	IF	1:400
c-Fos	Rabbit	Cell signaling, 2250S	IF	1:1000
Anti-rabbit IgG, Alexa 594	Donkey	Invitrogen, A21207	IF	1:500
Anti-mouse IgG, Alexa 488	Donkey	Invitrogen, A21202	IF	1:500
Anti-rabbit IgG, Alexa 405	Donkey	Invitrogen, A48258	IF	1:500
Anti-rat IgG, Alexa 594	Donkey	Invitrogen, A48271	IF	1:500
APC anti-mouse CD45	Mouse	BioLegend, 103112	Flow Cytometry	1:200
FITC anti-mouse/human CD11b	Mouse	Biolegend, 101206	Flow Cytometry	1:500
PerCP/Cyanine5.5 anti-mouse Ly-6G	Mouse	Biolegend, 127616	Flow Cytometry	1:200
APC-Cy7 anti-mouse TREM-2	Mouse	Bioss, bs-2723r	Flow Cytometry	1:400

### 2.6 Immunofluorescence

After they were deeply anesthetized, male mice were transcardially perfused with 0.9% cold saline (100 mL/100 g), followed by 4% PFA (100 mL/100 g). The brains were then postfixed with 4% PFA for 24 h at 4°C and cryoprotected with 30% sucrose at 4°C. Next, 30 µm thick cryosections were cut with a Leica microtome. Subsequently, frozen sections were permeabilized with 0.4% Triton X-100 in PBS and 10% normal donkey serum for 2 h at RT, followed by incubation overnight at 4°C with primary antibodies Iba1, CD68, SYP, PSD95, CaMKII, and c-Fos. After three washes in PBS, brain slices were incubated with secondary antibodies in a blocking buffer at RT for 2 h and then mounted on positively charged slides. Images were captured using confocal microscopy (Olympus, Tokyo, Japan).

### 2.7 Quantitative reverse transcription polymerase chain reaction

Prelimbic tissue samples were collected and total RNA extracted with a Spin Column Animal Total RNA Purification Kit (B518651-0100, Sheng-gong, China) following the manufacturer’s instructions. Total RNA was reverse-transcribed into cDNA in a 20 μL reaction volume using the Hiscript IIQ RTSuperMix for qPCR (R223-01, Vazyme, China). qRT-PCR was performed with the Thermo fly QuantStudio 7 Flex instrument (Roche Diagnostics, Switzerland) and SYBR Premix Ex TaqII kit (RR820A, Takara, Japan) and GAPDH or actin was used as an internal control. The relative expression ratio of every gene was normalized to control groups through the △Ct method (2^−△△Ct^). Primers sequences (Generay, China) are shown in **Table 3**.

**Table 3:** Experimental primers sequences used in this article.

Gene	Primary pair (5’-3’)	
GAPDH	Fw: AAGAAGGTGGTGAAGCAGG	Rw: GAAGGTGGAAGAGTGGGAGT
β-Actin	Fw: GGGAAATCGTGCGTGAC	Rw: AGGCTGGAAAAGAGCCT
TNF-α	Fw: GCAAAGGGAGAGTGGTCA	Rw: CTGGCTCTGTGAGGAAGG
IL-1β	Fw: TGGTGTGTGACGTTCCC	Rw: TGTCCATTGAGGTGGAGAG
IL-6	Fw: ACAGAAGGAGTGGCTAAGGA	Rw: AGGCATAACGCACTAGGTTT
Trem-2	Fw: TTGCTGGAACCGTCACC	Rw: GGGCACCCTCGAAACTC
P2ry12	Fw: ATTGACCGCTACCTGAAGAC	Rw: GCCTCCTGTTGGTGAGAAT
Cx3cr1	Fw: ACAAGCGAGGGAGATGG	Rw: GGTTGGTTTGCAGGCAT
Tmem119	Fw: CTGGTGCCAAGGCTGAC	Rw: GTGGTGCGTTAGGGTGAAG
Csf1r	Fw: TAACGCCGAAGTGGGAT	Rw: TATGCCAGCGTCTTGGA
ApoE	Fw: CGGCTCTCCACACACCT	Rw: CTCACGGATGGCACTCAC

### 2.8 Golgi-Cox staining and dendritic spine counting

Golgi-Cox staining was used to visualize neuronal morphometry. Brains were stained using a Golgi staining kit (1-250029-12, GenMed Scientifics Inc., USA) according to the manufacturer’s protocol. Briefly, brain tissues were immersed in the fix buffer for 2 weeks in the dark at RT. Then, the tissues were stored in 30% sucrose for another 3 days in the dark at 4°C. A cryotome (chamber temperature −22°C) was used to prepare 100 μm-thick sections. Images were obtained with an Olympus BX53, then used to analyze the dendritic spines within the prelimbic region.

### 2.9 Chemical administration

For microglia depletion, PLX5622 (Plexxikon), a selective CSF-1 receptor inhibitor, was added at a dose of 1.2 g PLX5622 per kilogram of diet for 14 days to achieve stable microglia depletion as we reported before (36). It has been additionally reported by other groups that microglia could be partially (300 mg PLX5622/kg food) or almost completely (1200mg/kg) ablated in mice (37, 38). Control mice were provided standard AIN-76 (Plexxikon) with the same base formula. PLX5622 was formulated in AIN-76A standard chow which was exposed to γ-rays irradiation during processing, and all mice received ad libitum access to food and water. To inhibit microglia activation, minocycline (Sigma, USA), was injected (i.p.) at dose of 40 mg/kg/day body weight 3 days before initial foot-shock exposure. Mice also received continual minocycline injections on the following days, until all behavioral sessions were conducted (39). Control group mice were treated with an equal volume of saline, following the same time course.

### 2.10 Flow cytometry and analysis

After collecting prelimbic tissue samples, the cells were stained with FITC anti-mouse/human CD11b antibody, APC anti-mouse CD45 antibody, PerCP/Cyanine5.5 anti-mouse Ly-6G antibody, and APC-Cy7 anti-mouse TREM-2 antibody. We assessed CD11b^+^CD45^int^Ly6G^−^ as microglia, CD11b^+^CD45^hi^Ly6G^−^ as macrophages, and CD11b^+^CD45^hi^Ly6G^hi^ as PMNs populations (3000 cells in total). All antibodies are listed in **Table 2**. All data were acquired on a FACS CantoII (BD Bioscience, USA), and analyzed using FlowJo software version X (BD Research, USA) to determine the percentage of each cell population and mean fluorescence intensity (MFI).

### 2.11 Microglia morphological analysis

To examine the morphology of Iba1-positive cells localized to the prelimbic area, we employed Sholl analysis, as previously described (33). Briefly, Z-stack images of individual microglia were obtained with the Olympus BX53 at a 60× oil immersion lens using 0.86 μm step size. The first shell was set at 10 μm, and following shells in 10 μm steps, to quantify the number of intersections in ImageJ (Fiji edition, NIH).

To assess CD68, PSD95, and SYP staining inside microglia, the IMARIS software (Imaris 9.0.1, Bitplane, Switzerland) was used to create a 3D reconstruction surface rendering. To ensure the microglial reconstruction process finished accurately, we established a threshold for subsequent analysis. Briefly, the volume of CD68 engulfed within Iba1 was calculated using the ‘surface’ function, and the number of PSD95 and SYP puncta engulfed within microglia and CD68 were reconstructed using the ‘Spots’ function in IMARIS. Two images were captured randomly per mouse, and each image had at least 10 cells, for 3 mice, in one group.

### 2.12 Statistical analysis

Data are represented as mean ± standard error of the mean (SEM). Unpaired Student’s t-tests were used to compare between groups. For multiple group comparisons, data were analyzed by one-way analysis of variance (ANOVA) or two-way ANOVA with Tukey’s multiple-comparison test for two-factorial designs. All analyses were performed using GraphPad Prism 8 (GraphPad Software, Inc., USA). Significance levels are displayed as *P < 0.05, **P < 0.01, ***P < 0.001.

## 3 Results

### 3.1 Fear conditioning promotes fear memory formation and glutamatergic neuronal activation in the prelimbic

**Figure 1:**
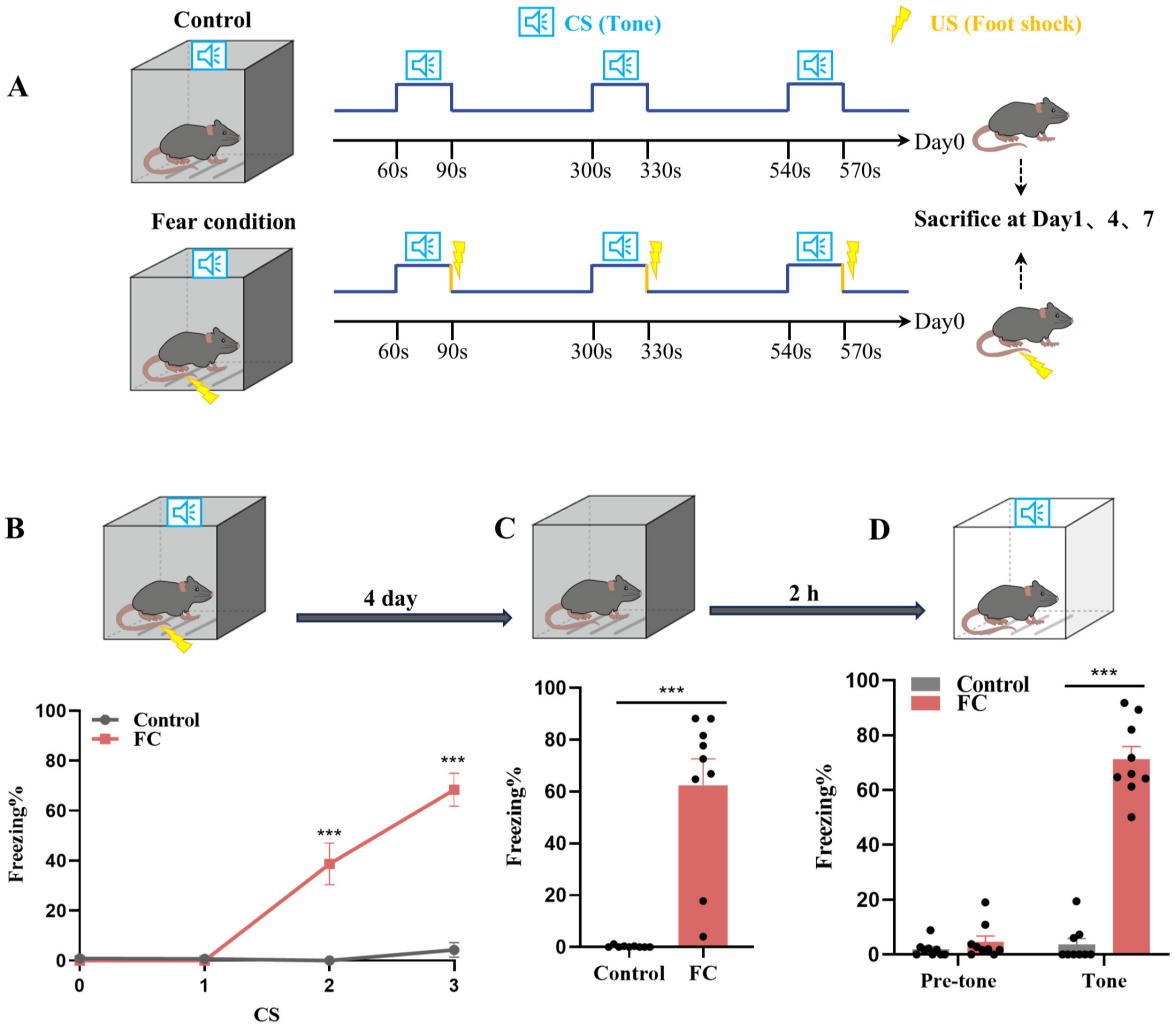
Fear conditioning-induced microglial activation and fear memory formation. **(A)** Experimental diagram of control and fear condition. **(B)** Freezing level during three CS–US pairings or control CS exposures (n = 9 mice, ***P < 0.001). **(C, D)** Freezing level during contextual **(C)** and cued **(D)** fear memory test compared with control mice (n = 9 mice, ***P < 0.001).

We used fear conditioning to model fear memory formation. As shown in **Figure 1A**, mice were exposed to paired sound (neutral stimulus) and electric foot-shock (aversive stimulus). To investigate microglia involvement in fear conditioning, we first observed the prelimbic area, which is involved in fear memory formation (7, 11). We then analyzed the microglia morphology of in the prelimbic tissue sections related to microglial activation status on day1, day4 and day7 after foot-shocks exposure. Immunofluorescence revealed that this fear conditioning exposure protocol induced significant microglia cell activation, characterized by decreased branchpoints and shorter processes, especially on day4 (**Supplementary Figures S1A, B**). Therefore, we set day4 as the key timepoint for further experiments. We next attempted to observed other brain regions, such as infralimbic and hippocampus, to evaluated the morphology of microglia. We found that microglia are significantly activated in infralimbic after 4 days exposed to fear condition, and also characterized by decreased branchpoints and shorter processes (**Supplementary Figures S1C, D**). Contrary to prelimbic and infralimbic, foot-shock did not impact microglial morphology in hippocampus compared to control group (**Supplementary Figures S1E, F**). To further define whether mice formed fear memory on day4, a fear conditioned model was used. During fear conditioning, mice exhibited a significantly increased freezing level (**Figure 1B**). On day4, mice also displayed higher freezing time percentages in both contextual fear memory (**Figure 1C**) and cued fear memory (**Figure 1D**) conditions compared with the control group.

**Figure 2:**
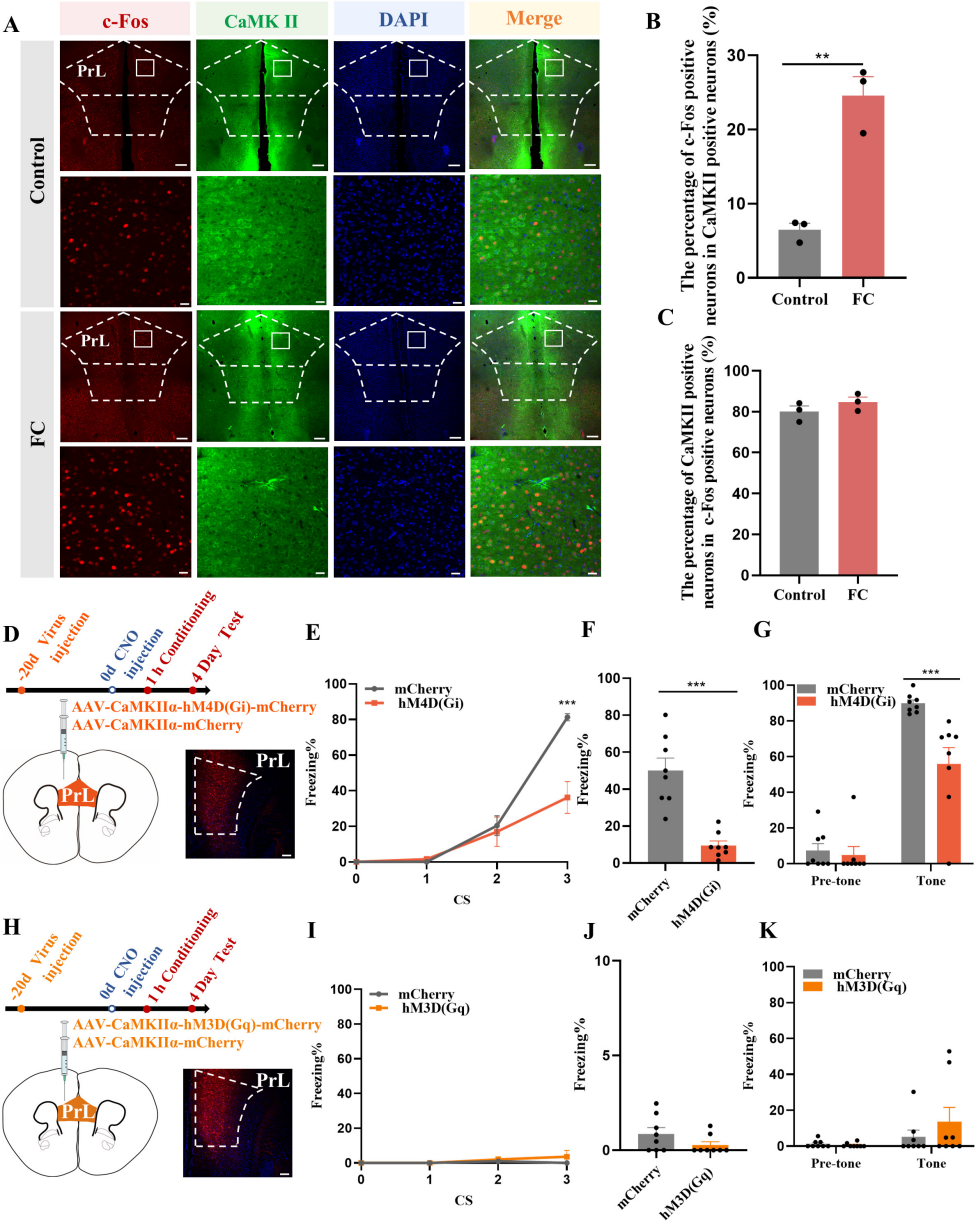
Activation of prelimbic glutamatergic neurons during fear conditioning. **(A)** Representative image of prelimbic c-Fos (red) and CaMKII (green) (scale bars = 200 μm [first and third rows]; 20 μm [second and fourth rows]). **(B)** Quantification of c-Fos^+^ proportion among CaMKII^+^ neurons (n = 3 mice, 2 slices per mouse, **P < 0.01). **(C)** Quantification of CaMKII^+^ proportion among c-Fos^+^ neurons (n = 3 mice). **(D)** Representative image illustrating prelimbic AAV expression (scale bars = 100 μm). **(E)** Percentage of freezing in mice with AAV-CaMKIIα-hM4D(Gi)-mCherry injection during fear conditioning compared with control mice (n = 8 mice, ***P < 0.001). **(F, G)** Percentage of freezing during contextual **(F)** and cued **(G)** fear memory test (n = 8 mice, ***P < 0.001). **(H)** Representative image illustrating prelimbic AAV expression (scale bars = 100 μm). **(I)** Percentage of freezing in mice with AAV-CaMKIIα-hM3D(Gq)-mCherry injection during fear conditioning compared with control mice (n = 8 mice). **(J, K)** Percentage of freezing during contextual **(J)** and cued **(K)** fear memory test (n = 8 mice).

Based on previous studies showing that activated microglia can influence neuronal activity through synaptic pruning (40), we concentrated on the role of activated microglia to detect whether they adjust neuronal activity in the fear memory formation process. In exploring prelimbic and infralimbic glutamatergic neuron excitability, immunofluorescence results showed that co-labeled c-Fos and CaMKIIα was not altered in infralimbic (**Supplementary Figures S2A, B**) but was higher in prelimbic compared to control group (**Figures 2A, B**). The percentage of prelimbic CaMKII-positive neurons among c-Fos-positive neurons was also >75%, suggesting that most of the neurons activated during fear conditioning were glutamatergic (**Figures 2A, C**). Moreover, we have showed negative staining controls in **Supplementary Figure S2C**.

To further evaluate the role of prelimbic glutamatergic neurons in fear memory formation, we microinjected the bilateral prelimbic area with viruses AAV-CaMKIIα-hM3D(Gq)-mCherry/AAV-CaMKIIα-hM4D(Gi)-mCherry or AAV-CaMKIIα-mCherry 21 days before behavioral tests (**Figures 2D, H**). Behavioral assessment showed that the percentage of freezing time was decreased in the hM4D(Gi) group compared to that in control group, as indicated that inhibition of glutamatergic neurons in the prelimbic reduced fear formation at 1 h after CNO intraperitoneal injection (**Figures 2E–G**). However, without fear training, activating glutamatergic neurons in the prelimbic under physiological condition did not impact the freezing level in behavioral test behavioral test performance (**Figures 2I–K**). Overall, these results indicate that prelimbic glutamatergic neurons were hyperactivated, and that chemogenetically inhibiting this hyperactivation decreased the level of freezing during fear memory formation. While just activating glutamatergic neurons without fear training makes no sense to fear formation.

### 3.2 Fear memory formation drives increased microglial phagocytosis, enabling microglia prelimbic synapse pruning

**Figure 3:**
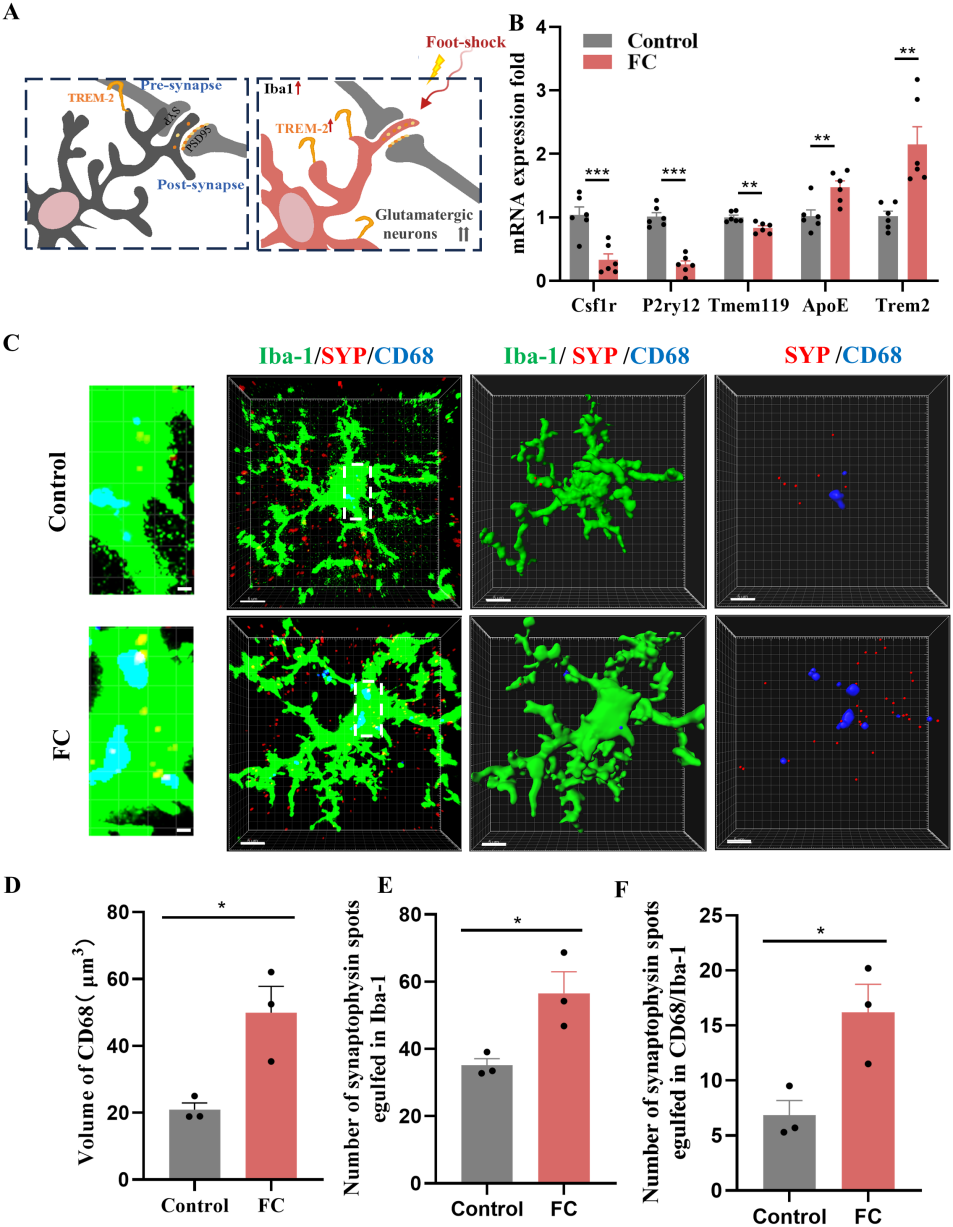
Fear memory formation drives increased microglial phagocytosis. **(A)** Schematic diagram showing fear conditioning-induced microglial activation and enhanced microglial capacity for engulfing synapse materials. **(B)** Relative mRNA expression in the prelimbic by qRT-PCR (n = 6 mice, **P < 0.01, ***P < 0.001). **(C)** Detailed figure (first row) and representative confocal (second row) and Imaris (middle and bottom rows) images of Iba1 (green), CD68 (blue), and SYP (red) from control and fear conditioned mice. Third row: reconstruction of Iba1 and CD68 staining. Fourth row: reconstruction of CD68 and SYP staining inside microglia. **(D)** CD68 volume inside microglia. **(E)** SYP puncta inside microglia. **(F)** SYP puncta inside CD68 phagosomes (n = 3 mice, 10 cells per mouse, *P < 0.05, scale bars = 5 μm and scale bars = 1 μm in detailed figure of the top row).

Microglia are required for synapse remodeling in the CNS, and exacerbating microglial activation leads to decreased excitatory synapses (41). As shown in **Supplementary Figures S1A, B**, several activated microglia were detected in the prelimbic area. Meanwhile, “homeostatic” is used as a technical term for referring microglia in physiological conditions, Hickman SE et al. defined the apparatus which microglia utilize to implement these homeostatic functions including P2ry12, Tmem119 and Csf1r (42). Moreover, these apparatuses have been proposed to play crucial roles via sensing of chemokines and cytokines, purinergic molecules, inorganic substances, changes in pH and amino acids. In our study, we found that the expression of P2ry12, Tmem119, and Csf1r were decreased while Trem2 and ApoE were heightened which indicated that microglia in our study is not like in a homeostatic state (**Figures 3A, B**). Furthermore, t-test analyses revealed a decreased proinflammatory cytokine IL-1, while mRNA levels of TNF-α and IL-6 were unaltered by foot-shock (**Supplementary Figure S3A**). Taken together, our results show that fear conditioning induced fear memory and microglia activation on day4, and emphasize the significant increased between control and FC microglial cells, several of which involve genes that promote homeostasis and phagocytosis but not genes that facilitate inflammation.

**Figure 4:**
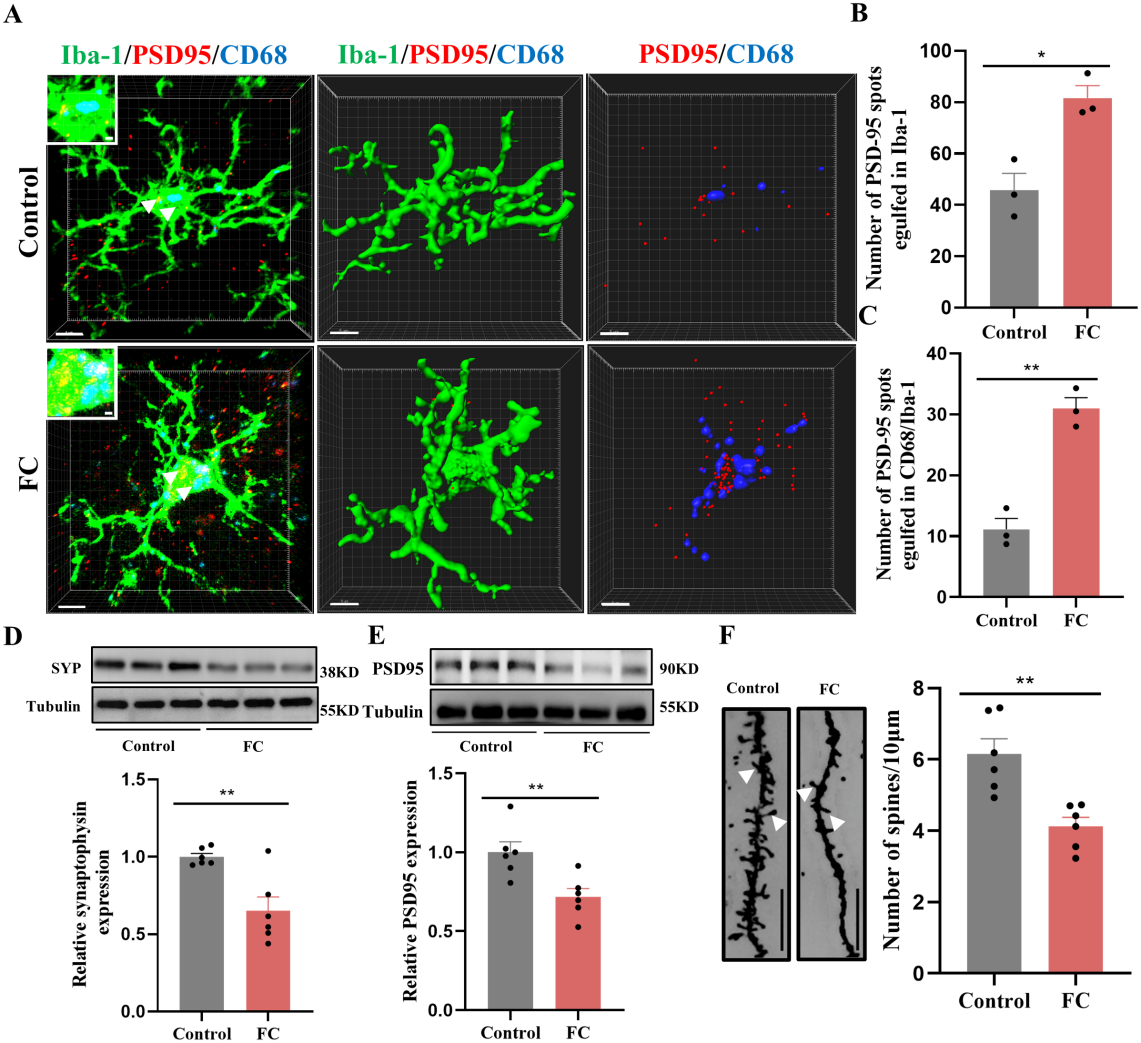
Fear memory formation drives increased microglial phagocytosis. **(A)** Representative confocal (top row) and Imaris (middle and bottom rows) images of Iba1 (green), CD68 (blue), and PSD95 (red) from control and fear conditioned mice. Middle row: reconstruction of Iba1 and CD68 staining. Bottom row: reconstruction of CD68 and PSD95 staining inside microglia. **(B)** PSD95 puncta inside microglia. **(C)** and PSD95 puncta inside CD68 phagosomes (n = 3 mice, 10 cells per mouse. *P < 0.05, **P < 0.01, scale bars = 5 μm and scale bars = 1 μm in detailed figure). **(D, E)** Protein level of prelimbic SYP and PSD95 detected by WB, and quantitative results of WB analyses (n = 6 mice, **P < 0.01). **(F)** Representative dendritic spine density of prelimbic neurons, and dendritic spine density quantification (n = 6 mice, 3 dendritic segments per mouse, **P < 0.01, Scale bars = 10 μm).

To directly confirm the capacity of microglia to phagocytose synaptic material, we assessed the effects of fear condition on lysosome volume (CD68 staining) and the number of presynaptic markers SYP or PSD95 puncta located inside microglia. We found that prelimbic CD68 volume (**Figures 3C, D**), number of SYP+ puncta inside microglia (**Figure 3E**), and number of SYP+ puncta inside CD68 (**Figure 3F**) were significantly increased in mice exposed to foot-shock (see 3D surface rendering in **Supplementary Videos 1**, **2**). Similar patterns were seen for the number of PSD95+ puncta inside microglia (**Figures 4A, B**) and the number of PSD95+ puncta inside CD68 (**Figure 4C**, **Supplementary Videos 3**, **4**). Biochemical assessment further indicated that fear formation decreased, in a microglia-mediated manner, the expressions of prelimbic SYP and PSD95, showing that increased microglial phagocytic capacity leads to prelimbic synapse loss after fear memory formation (**Figures 4D, E**). Additionally, deceased spine density in the fear conditioned group (**Figure 4F**) was confirmed by Golgi-Cox staining. Overall, these findings reveal that fear memory formation results in increased microglial phagocytosis, enabling microglia to prune prelimbic synapses.

### 3.3 Pharmacological depletion of microglia protects against excessive synaptic pruning and fear memory formation

**Figure 5:**
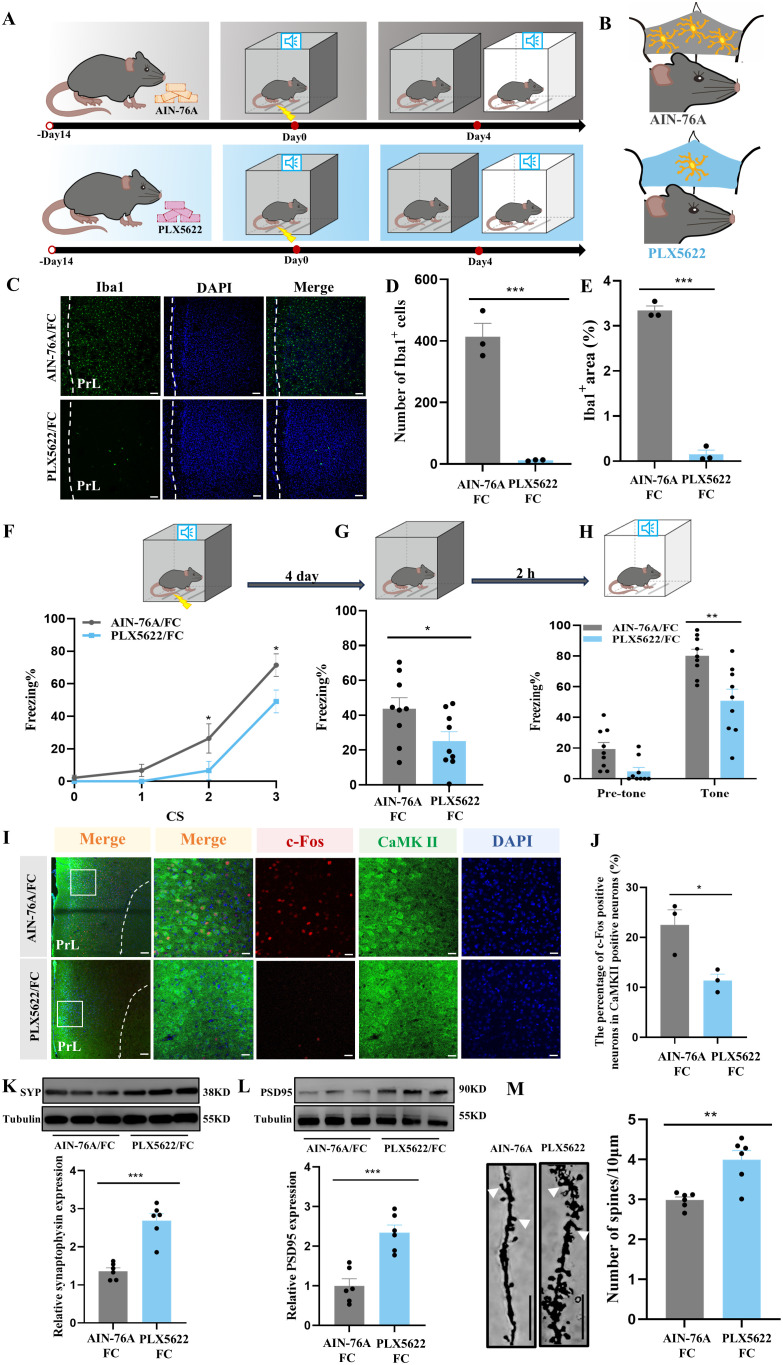
Ablation of microglia with PLX5622 protects against increased microglial phagocytosis and formation of fear memory. **(A)** Time course for PLX5622 and AIN-76A administrations and behavior tests. **(B)** Schematic diagram showing PLX5622 efficacy. **(C)** Representative confocal images of Iba1 immunostaining (green) of prelimbic microglia of mice with PLX5622 and AIN-76A feeding (scale bars = 100 μm). **(D, E)** Quantification of Iba1^+^ cells and Iba1^+^ area (n = 3 mice, 2 slices per mouse, ***P < 0.001). **(F)** Percentage of freezing in mice with control or PLX5622 feeding during fear conditioning (n =9 mice, *P < 0.05). **(G, H)** Percentage of freezing in control or PLX5622 feeding mice during contextual **(G)** and cued **(H)** fear memory test (n =9 mice, *P < 0.05, **P < 0.01). **(I)** Representative images of prelimbic c-Fos (red) and CaMKII (green) in mice with PLX5622 and AIN-76A feeding (scale bars = 100 μm [first row]; 20 μm [the other rows]). **(J)** Number of prelimbic CaMKII and c-Fos double-labeled neurons (n = 3 mice, 2 slices per mouse, *P < 0.05). **(K, L)** Protein level of prelimbic SYP and PSD95 detected by WB, and quantitative results for mice treated with or without PLX5622 feeding (n = 6 mice, ***P < 0.001). **(M)** Representative prelimbic neuron dendritic spine density, and dendritic spine density quantification for mice treated with or without PLX5622 feeding (n = 6 mice, 3 dendritic segments per mouse, **P < 0.01, scale bars = 10 μm).

To further interrogate the synaptic pruning effects of microglia in fear memory formation, mice were treated for two weeks with PLX5622, a selective CSF-1 receptor inhibitor reported to effectively deplete microglia, added to the standard AIN-76A chow (**Figures 5A, B**). Results from immunostaining showed obvious microglia depletion in prelimbic of PLX5622-treated mice (**Figures 5C–E**). We also found that, compared with the AIN-78A group, mice in the microglial deletion group displayed reduced freezing during fear conditioning and contextual and cued recall tests (**Figures 5F–H**).

Of note, we assessed the changes in number of co-labeled c-Fos and CaMKIIα neurons in PLX5622- and AIN-76A-treated mice, to determine the influence of microglia in neuronal activity. Prelimbic glutamatergic neuron activity was lower in the PLX5622-treated group than in the AIN-76A-treated group (**Figures 5I, J**). To examine whether microglia have an impact on fear-induced PSD95 and SYP protein levels as shown in **Figures 3K, L**, respectively, we used WB to analyze prelimbic SYP and PSD95 expressions in animals treated with PLX5622. There was a significant effect of microglia on protein levels, with PLX5622 group mice displaying a robust increase in SYP and PSD95 compared with AIN-76A mice (**Figures 5K, L**). Furthermore, the spine density also increased with inhibitor treatment (**Figure 5M**). These findings support the possibility that microglia depletion protects against fear formation by regulating neuronal activity to reduce excessive synaptic pruning.

### 3.4 Minocycline-inhibited microglia activation protects against excessive synaptic pruning and fear memory formation

**Figure 6:**
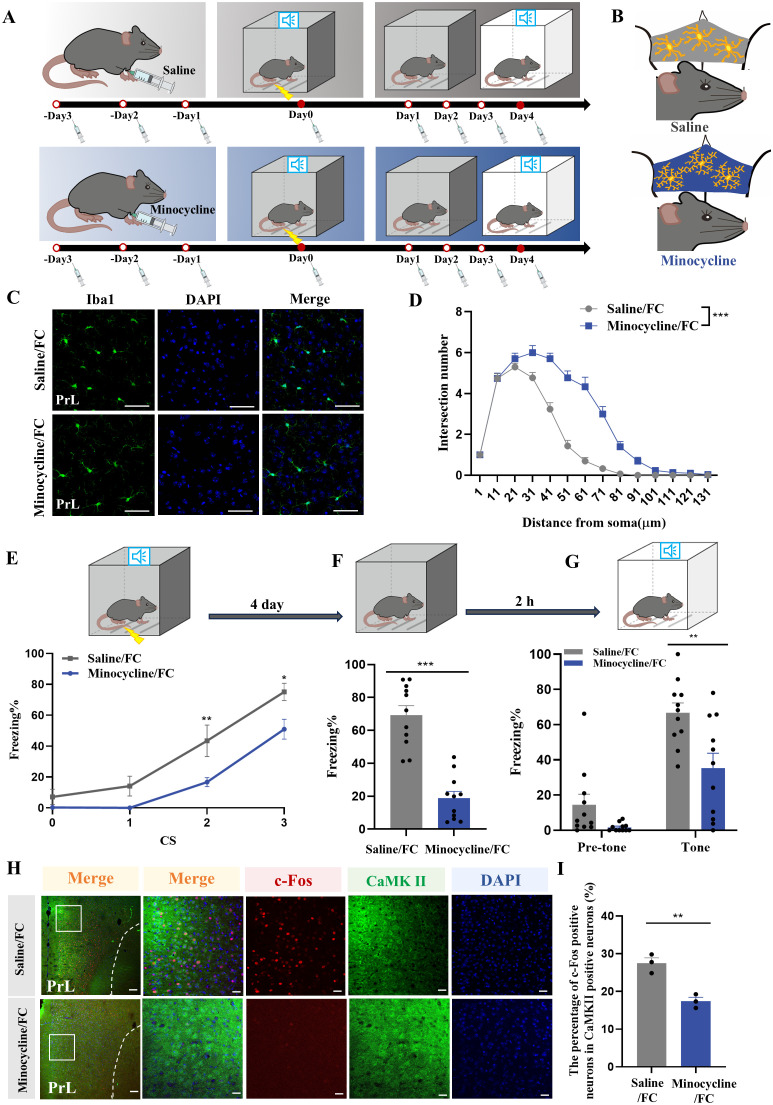
Microglia inhibition with minocycline protects against increased neuronal activity and fear memory formation. **(A)** Time course for i.p. minocycline or saline injections and behavior tests. **(B)** Schematic diagram showing minocycline efficacy. **(C)** Representative confocal images of Iba1 immunostaining (green) of microglia in the prelimbic of mice with minocycline injection (scale bars = 50 μm). **(D)** Sholl analysis of microglial morphology of mice with or without minocycline (n = 3 mice, 10 cells per mouse, ***P < 0.001). **(E)** Percentage of freezing in control or minocycline-treated mice during fear conditioning (n = 11 mice, *P < 0.05, **P < 0.01). **(F, G)** Percentage of freezing in control or minocycline-treated mice during contextual **(F)** and cued **(G)** fear memory test (n =11 mice, **P < 0.01, ***P < 0.001). **(H)** Representative image of prelimbic c-Fos (red) and CaMKII (green) of mice with minocycline microinjection (scale bars = 100 μm [first row]; 20 μm [the other rows]). **(I)** Number of prelimbic CaMKII and c-Fos double-labeled neurons (n = 3 mice, 2 slices per mouse, **P < 0.01).

To explore the effects of microglial activation on fear memory, minocycline, a drug which inhibits microglial activation, was administered via i.p. injection (**Figures 6A, B**). We evaluated prelimbic cortex microglial morphology after foot-shock, finding that minocycline significantly suppressed microglial activation, characterized by increased branchpoints and longer processes (**Figures 6C, D**). This treatment also decreased the fear response from fear conditioning and improved performance in contextual and cued fear memory acquisition (**Figures 6E–G**). Consistent with this, the effects of minocycline on behavior tests were reproduced by prelimbic microinjection (**Supplementary Figures S4A–C**), and immunofluorescence showed decreased level of neuronal activity of glutamatergic neurons after inhibiting the activation of microglia in the minocycline group (**Figures 6H, I**).

**Figure 7:**
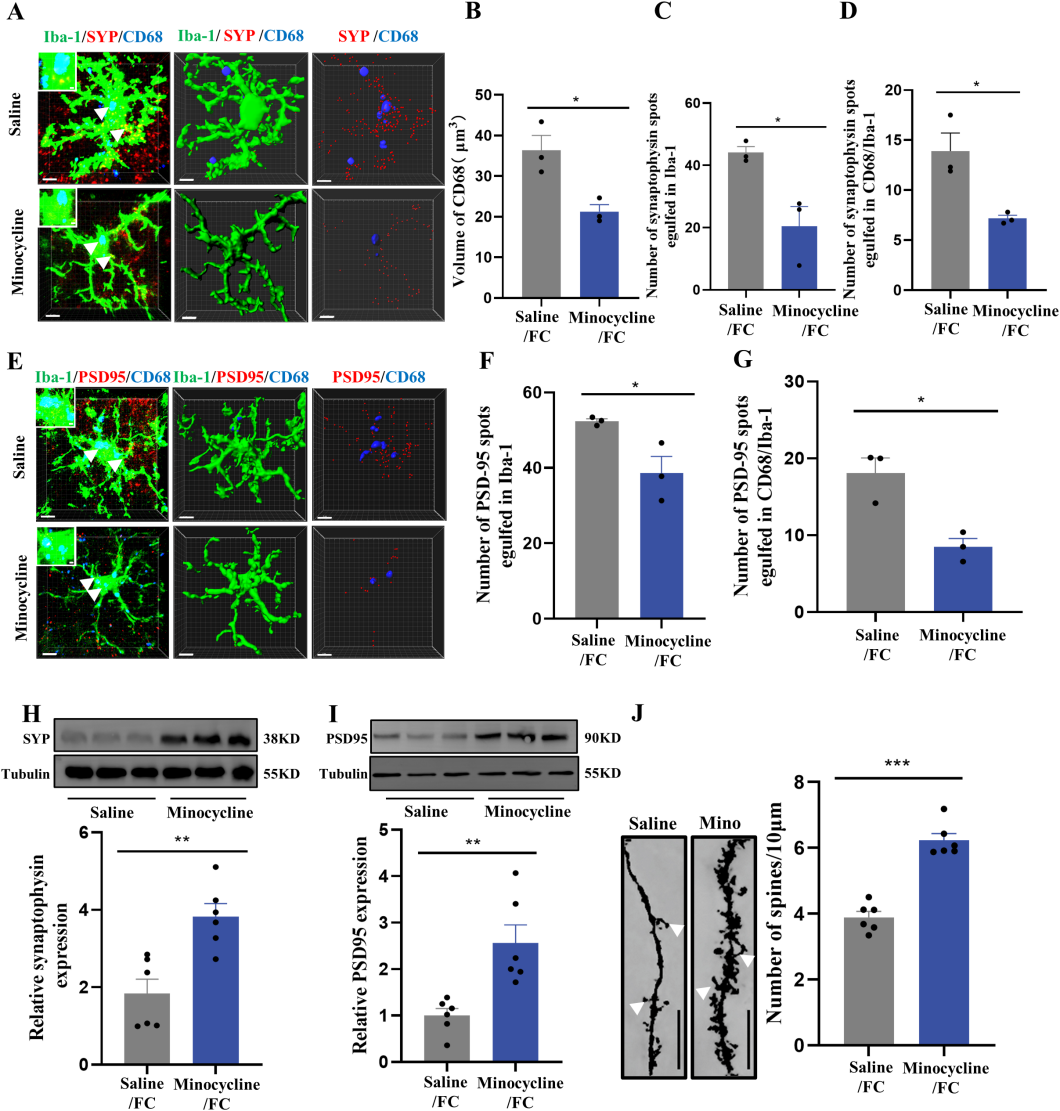
Inhibition of microglia with minocycline protects against increased microglial phagocytosis. **(A)** Representative confocal (top row) and Imaris (middle and bottom rows) images of Iba1 (green), CD68 (blue), and SYP (red) from saline- and minocycline-treated mice. Middle row: reconstruction of Iba1 and CD68 staining. Bottom row: reconstruction of CD68 and SYP staining inside microglia. **(B)** CD68 volume inside microglia. **(C)** SYP puncta inside microglia. **(D)** SYP puncta inside CD68 phagosomes (n = 3 mice, 10 cells per mouse, *P < 0.05, scale bars = 5 μm and scale bars = 1 μm in detailed figure). **(E)** Representative confocal (top row) and Imaris (middle and bottom rows) images of Iba1 (green), CD68 (blue), and PSD95 (red) from saline- and minocycline-treated mice. Middle row: reconstruction of Iba1 and CD68 staining. Bottom row: reconstruction of CD68 and PSD95 staining inside microglia. **(F)** PSD95 puncta inside microglia. **(G)** PSD95 puncta inside CD68 phagosomes (n = 3 mice, 10 cells per mouse, *P < 0.05, scale bars = 5 μm and scale bars = 1 μm in detailed figure). **(H, I)** Protein levels of prelimbic SYP and PSD95 detected by WB, and WB quantification (n = 6 mice, **P < 0.01). **(J)** Representative prelimbic neuron dendritic spine density, and dendritic spine density quantification (n = 6 mice, 3 dendritic segments per mouse, ***P < 0.001, scale bars = 10 μm).

Next, we used immunofluorescence to analyze prelimbic tissue sections for SYP+ or PSD95+ puncta within CD68 and microglia. Confocal imaging coupled with 3D cell surface rendering showed that the CD68 volume (**Figures 7A, B**), number of SYP+ puncta inside microglia (**Figure 7C**), and number of SYP+ puncta inside CD68 (**Figure 7D**) were all decreased in the minocycline-treated group (see 3D surface rendering in **Supplementary Videos 5**, **6**). Similar findings were revealed in PSD95+ puncta (**Figures 7E–G**) (see 3D surface rendering in **Supplementary Videos 7**, **8**). WB for prelimbic SYP and PSD95 further confirmed these findings. SYP and PSD95 protein levels increased with minocycline treatment, revealing an obvious decrease in microglial phagocytosis (**Figures 7H, I**). Golgi-Cox staining results also revealed that minocycline treatment reduced spine density reductions (**Figure 7J**). These collective findings suggest that microglial activation contributes to fear formation-related behaviors, apparently via increased foot shock-primed engulfment of prelimbic glutamatergic neuronal synapses by microglia.

### 3.5 TREM2 is associated with increased microglial phagocytosis, and TREM2 deficiency attenuates fear memory formation

**Figure 8:**
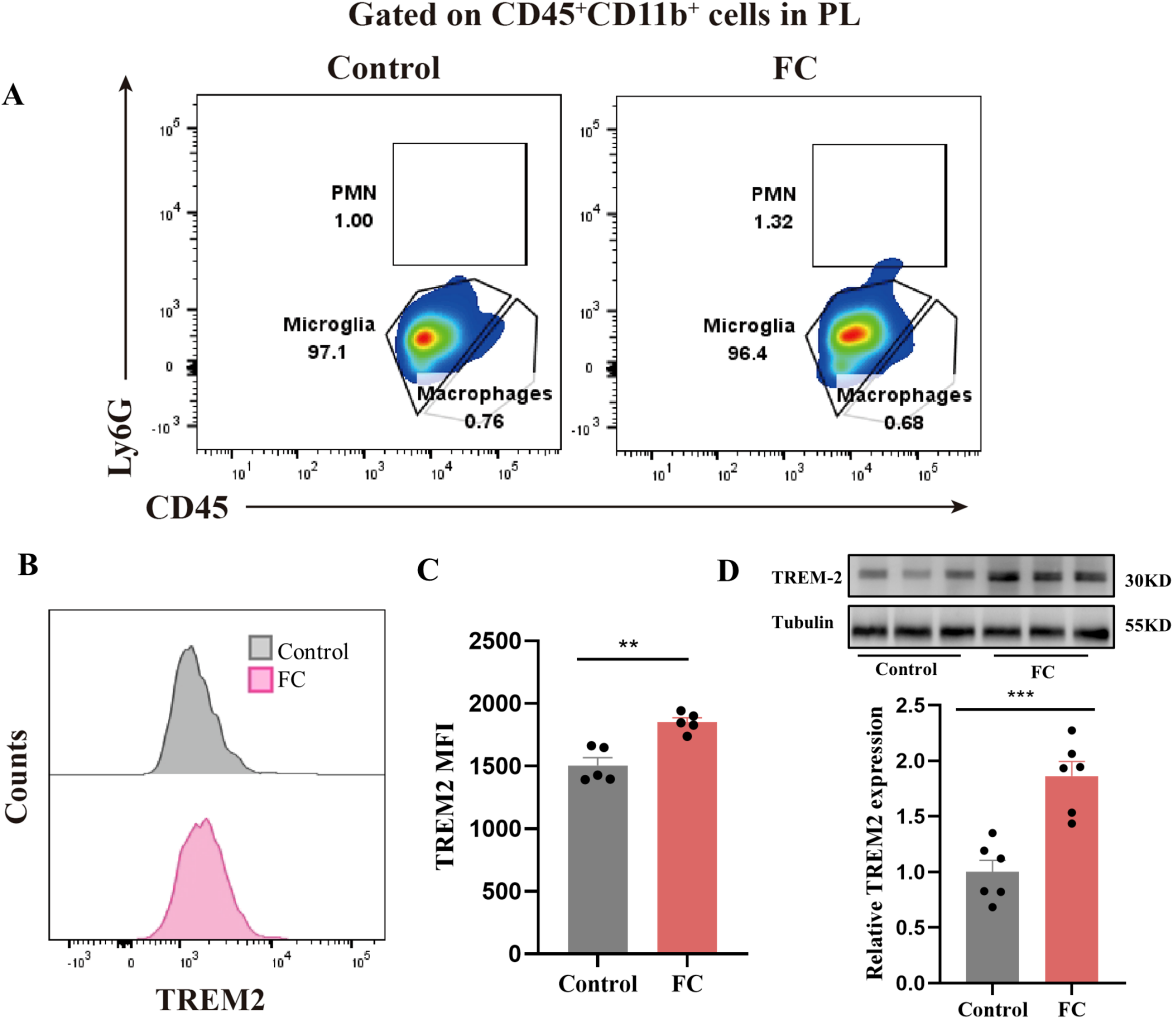
Prelimbic TREM2 expression is upregulated after foot-shock exposure. **(A)** Representative plots of CD11b^+^CD45^int i^Ly6G^−^microglia, CD11b^+^CD45^hi^Ly6G^−^ macrophages, and CD11b^+^CD45^hi^Ly6G^hi^ PMNs populations (3000 cells in total) after foot-shock exposure. **(B)** Representative histograms of microglial TREM2 expression. **(C)** Prelimbic microglial TREM2 MFI (n = 5 mice, **P < 0.01). **(D)** Prelimbic TREM2 protein level detected by WB, and WB quantification (n = 6 mice, ***P < 0.001).

Previous studies have demonstrated that TREM2 is vitally important to microglial phagocytosis during brain development and neurodegenerative diseases (43–45). To investigate the possible involvement of TREM2 in fear-induced excessive synaptic pruning by microglia, flow cytometry staining of CD11b^+^CD45^int^ microglia to detect TREM2 expression on microglia revealed a markedly increased TREM2 MFI in the fear conditioned group (**Figures 8A–C**). Consistent with these data, TREM2 expression was assessed by WB. Prelimbic TREM2 protein in WT animals exposed to foot-shock was increased compared with that in control mice (**Figure 8D**). Thus, we speculate that microglial TREM2 upregulation may be involved in fear memory formation through enhanced microglial phagocytosis.

**Figure 9:**
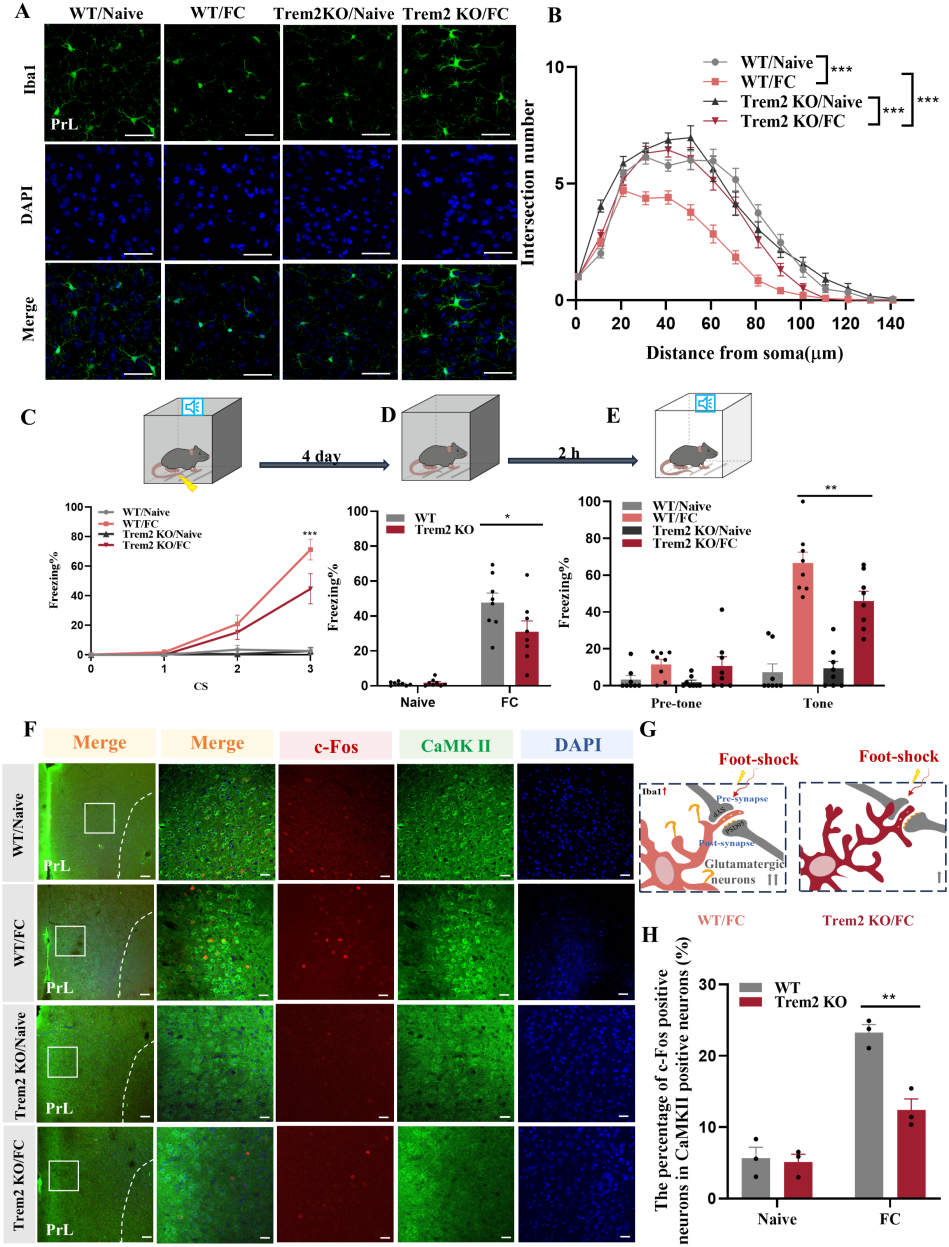
TREM2 inhibition affects neuronal activity and fear memory formation. **(A)** Representative confocal images of prelimbic microglia Iba1 immunostaining (green) in Trem2 KO mice (scale bars = 50 μm). **(B)** Sholl analysis of microglial morphology (n = 3 mice, 10 cells per mouse, ***P < 0.001). **(C)** Percentage of freezing in Trem2 KO mice during fear conditioning (n = 8 mice, ***P < 0.001). **(D, E)** Percentage of freezing during contextual **(D)** and cued **(E)** fear memory test (n = 8 mice, *P < 0.05, **P < 0.01). **(F)** Representative image of prelimbic c-Fos (red) and CaMKII (green) in Trem2 KO mice (scale bars = 100 μm [first row]; 20 μm [the other rows]). **(G)** Schematic diagram showing that TREM2 inhibition protects against prelimbic glutamatergic neuron activation after exposed to foot-shock. **(H)** Number of prelimbic CaMKIIα and c-Fos double-labeled neurons (n = 3 mice, 2 slices per mouse, **P < 0.01).

As shown in **Figures 9A, B**, microglia of Trem2 KO mice exhibited increased branchpoints and longer processes compared with WT mice after foot-shock exposure, while there were no significant differences between these groups without fear condition. These results demonstrate that Trem2 KO effectively inhibited microglial activation, which could not rebound to pre-activation levels. To gain further insights into whether TREM2 is directly involved in fear memory, WT and Trem2 KO mice were exposed to the fear conditioning paradigm, and contextual and cued fear memory tests. In the normal condition (without foot-shock), Trem2 KO mice showed unaltered behavioral fear responses compared with WT mice (**Figures 9C–E**). However, two-way ANOVA on fear response after foot-shock exposure revealed significantly decreased fear memory formation in Trem2 KO mice, suggesting that the KO of Trem2 impaired the fear memory formation process. These results were replicated by prelimbic microinjection of Trem2 siRNA or control RNA (**Supplementary Figures S5A–C**). Staining analysis of prelimbic c-Fos and CaMKIIα (**Figures 9F–H**) revealed that, compared with WT mice, neuronal activity in Trem2 KO mice was reduced after foot-shock exposure, with no obvious differences in Trem2 KO and WT mice without foot-shock exposure.

**Figure 10:**
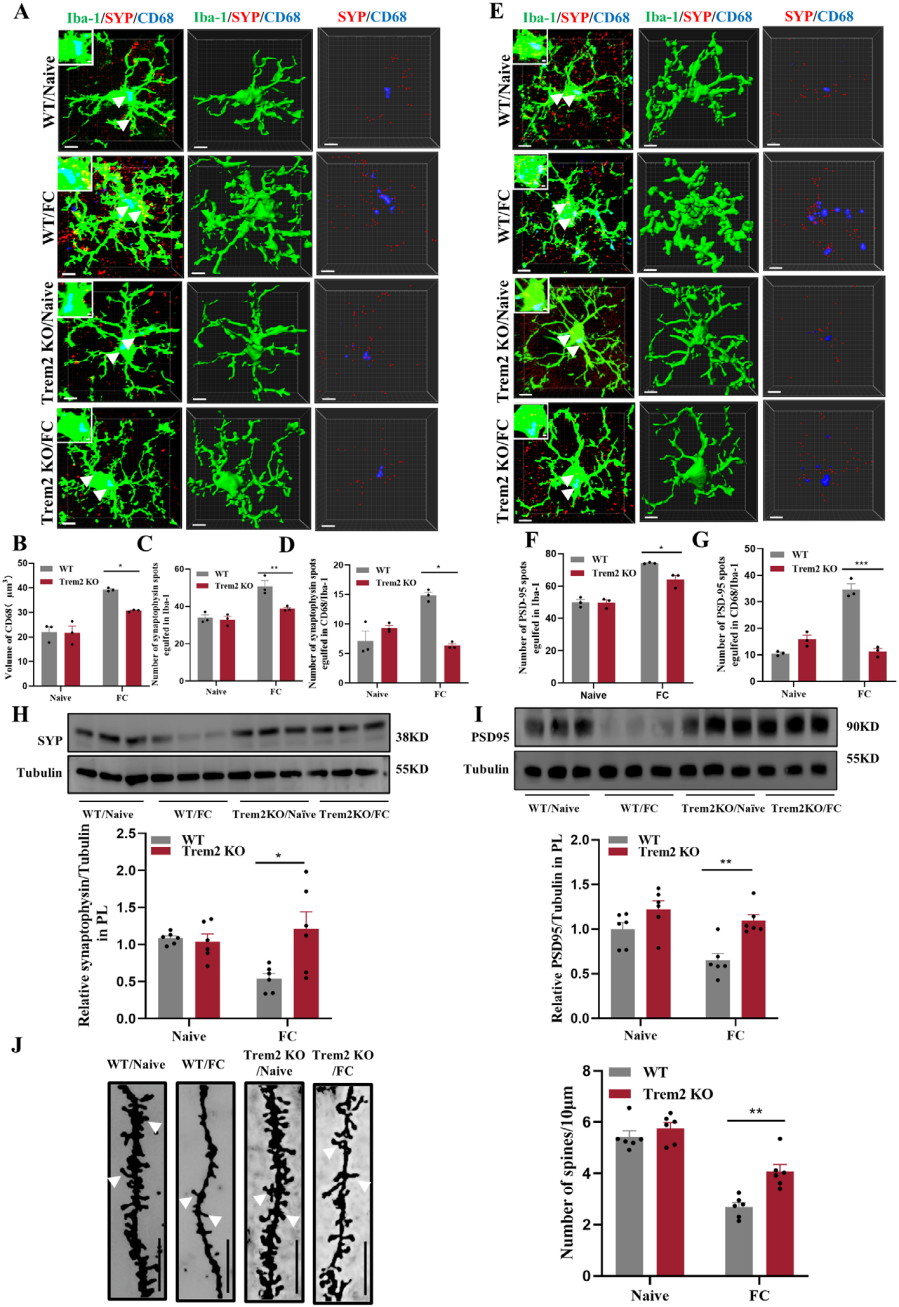
TREM2 inhibition protects against increased microglial phagocytosis. **(A)** Representative confocal (top row) and Imaris (middle and bottom rows) images of Iba1 (green), CD68 (blue), and SYP (red) from WT and Trem2 KO control mice and WT and Trem2 KO mice with three CS–US pairings exposure. Middle row: reconstruction of Iba1 and CD68 staining. Bottom row: reconstruction of CD68 and SYP staining inside microglia. **(B)** CD68 volume inside microglia. **(C)** SYP puncta inside microglia. **(D)** SYP puncta inside CD68 phagosomes (n = 3 mice, 10 cells per mouse, *P < 0.05, **P < 0.01, scale bars = 5 μm and scale bars = 1 μm in detailed figure). **(E)** Representative confocal (top row) and Imaris (middle and bottom rows) images of Iba1 (green), CD68 (blue), and PSD95 (red). Middle row: reconstruction of Iba1 and CD68 staining. Bottom row: reconstruction of CD68 and PSD95 staining inside microglia. **(F)** PSD95 puncta inside microglia. **(G)** PSD95 puncta inside CD68 phagosomes (n = 3 mice, 10 cells per mouse, *P < 0.05, ***P < 0.001, scale bars = 5 μm and scale bars = 1 μm in detailed figure). **(H, I)** Protein level of prelimbic SYP and PSD95 detected by WB, and quantitative WB results (n = 6 mice, *P < 0.05, **P < 0.01). **(J)** Representative prelimbic neuron dendritic spine density, and dendritic spine density quantification (n = 6 mice, 3 dendritic segments per mouse, **P < 0.01, scale bars = 10 μm).

We next performed 3D cell surface rendering to demonstrate the potential underlying mechanism through which microglial activation may impact synaptic pruning in the prelimbic. We found that CD68 volume (**Figures 10A, B**), number of SYP+ puncta inside microglia (**Figure 10C**) and the number of SYP+ puncta inside CD68 (**Figure 10D**) located in Trem2 KO mice, which exposed to electric foot-shock, were all decreased compared with WT mice (see 3D surface rendering in **Supplementary Videos 9–12**). Meanwhile, in line with such changes in SYP+ puncta, PSD95+ puncta (**Figures 10E–G**) were decreased in Trem2 KO group as well (see 3D surface rendering in **Supplementary Videos 13-16**). Replicating these immunofluorescence results, WB revealed that SYP and PSD95 protein levels (**Figures 10H, I**) also improved with Trem2 KO in the prelimbic. In addition, we analyzed the density of dendritic spines on prelimbic neurons using Golgi-Cox staining. As the results have shown, Trem2 knockout increased the density of the dendritic spine after foot-shock (**Figure 10J**). These cumulative findings demonstrate that prelimbic microglial TREM2 may be involved with regulating microglia-mediated excessive synaptic pruning and behavioral performance on fear memory formation tests.

## 4 Discussion

In past research, the neurobiological mechanisms of PTSD have been studied extensively, and some key brain structures, including the medial prefrontal cortex, hippocampus and amygdala were identified. Herein, we revealed that microglia in the prelimbic and infralimbic were notably activated, characterized by decreased branchpoints and shorter processes, especially on day4. However, microglia in the hippocampus showed similar morphological characteristics compared with the fear conditioning group. From the Li et al. study revealed that microglia in the amygdala showed no significant changes in number or morphology, and only subtle increases in process length in proximal (10µm) and distal processes (40µm) from the microglial cell soma in the basolateral amygdala (39). Nevertheless, our research group previously demonstrated that inhibition of glutamatergic neurons in the basolateral amygdala was unaltered in the fear learning progress, but decreased the freezing level in fear extinction (35).

Mice in this study were exposed to electric foot-shock (1 mA × 1 s × 3 times) to induce conditioned fear memory. Other rodent models of PTSD symptoms include the single prolonged stress model (46), predator stress (47) and chronic variable stress (48), among these, foot-shock is common to mouse experiments. This is both because the freezing response is modulated according to repeated foot-shock (49), and because fear conditioning mimics more PTSD symptoms (i.e., including anxiety and extinction deficiency) (35). Behavioral effects suggesting that this fear formation mouse model was successful include that freezing time was much longer in the foot-shock exposure group compared with the control group. In this paradigm, animals pair a neutral CS (e.g., sound, light) with an aversive US (e.g., electric shock) to form the fear memory. Furthermore, fear conditioning involves specific fear memory formation stages, including fear memory acquisition, consolidation, and reconsolidation. Mice underwent CS–US pairings during fear memory acquisition, then transferred this newly acquired memory into stable memory via fear memory consolidation, and further processing the formed fear memory through fear memory reconsolidation (50).

**Figure 11:**
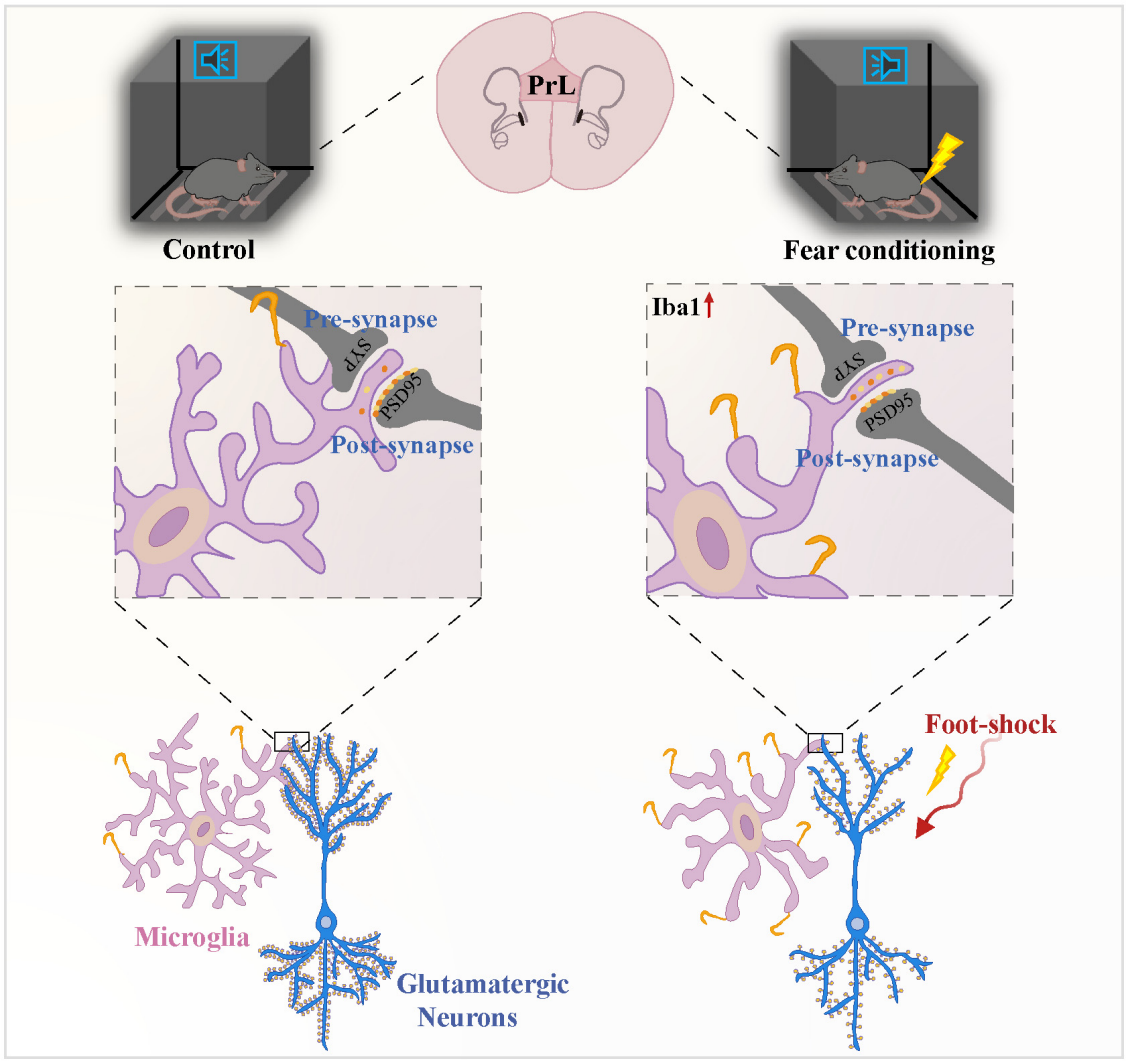
Microglial TREM2 participates in fear memory formation through excessive prelimbic cortex synaptic pruning. Fear conditioning induced microglial activation and enhanced microglial capacity for engulfing synapse materials, which are mainly on glutamatergic neurons. TREM2 also served as a key microglial regulator, leading to increasing synaptic pruning.

Microglial states are dynamic whereby microglia are capable of responding to different disease progression through changing their molecular profile, morphology, and ultrastructure, as well as motility and function (51). Many various and context-dependent microglial states have been named in different species and models including disease-associated microglia (DAMs) (52), microglial neurodegenerative phenotype (MGnD) (53), activated response microglia (ARMs) and interferon-responsive microglia (IRMs) (54), human AD microglia (HAMs) (54); microglia inflamed in multiple sclerosis (MS) (MIMS) (55); lipid-droplet-accumulating microglia (LDAMs) (56) and glioma-associated microglia (GAMs) (57). The DAMs is firstly described as a protective microglia cluster related to neurodegenerative diseases in AD transgenic (52). Now more researchers have identified DAMs as a complex core signature which are advantageous in certain contexts may be disadvantageous in others although in same states, strictly depending on the complicated interactions between microglia and their surrounding environment (58). A set of genes associated with the DAMs signature were explored including an upregulation of TREM2, ApoE, CD11c, CLEC7A, and LPL and downregulation of TGFβ, Csf1r, P2ry12, and Tmem119 (59–61). In line with previous study, we identified an upregulation of Trem2 and ApoE and downregulation of P2ry12, Tmem119, and Csf1r. Microglia serve as a major contributing factor in PTSD pathogenesis, potentially leading to synaptic dysfunction and neuronal impairment (62–64). Previous work in mice has demonstrated that PTSD promotes structural and proteomic changes in microglia, which may serve as a direct link between microglial activation and mental disorders. Significantly, inhibiting or depleting microglia decreased PTSD-like behaviors in mice (39). Activation of the NLRP3 inflammasome may play an injurious role in the PTSD development progress, while Nlrp3 knockout obviously alleviated both microglial activation and increased freezing time (31). There is evidence in PTSD patients that intense psychological stress induces activation of microglia in certain brain regions related to threat appraisal and emotional responses, and alters their phenotypic and functional properties through sterile neuroinflammatory pathways (65, 66). Preclinical models of have PTSD revealed that microglial cells are temporally and spatially altered during the PTSD development, and this has been associated with the release of proinflammatory mediators and cytotoxic factors (67, 68). In line with these results, post-mortem research in PTSD patients suggested that not only is the expression of pro-inflammatory cytokine IL-1α secreted by activated microglia decreased in the dorsolateral prefrontal cortex (DLPFC) (69), expressions of microglia-related genes were decreased in females with PTSD (70). Herein, we employed various techniques such as immunofluorescence staining, 3D cell surface rendering, and behavior testing to identified microglial participation in synaptic pruning and prelimbic glutamatergic neuronal activity, revealing that TREM2 may alter activated microglia-mediated synaptic engulfment, resulting in formatting the fear memory in mice after exposed to foot-shock (**Figure 11**).

Synaptic pruning is a process by which microglia dynamically eliminate unnecessary synapses during brain development or specific diseases (71, 72). The tendency to prune less active synapses, and to advance stronger or more active synapses, causes neuronal circuits to become more sophisticated (72). This is in line with the report that axon and dendrite pruning is pointed to make contributions to the establishment of advanced connections and is instrumental for normal brain development (73). In addition, pharmacologic depletion of microglia during postnatal days 2–13 by CSF1R disrupted the synaptic pruning process and led to polyinnervated neurons in the medial nucleus of the trapezoid body, rather than healthy monoinnervated neurons (74). During development of the retinogeniculate system in binocular animals, complement protein C1q and the downstream complement protein C3 labeled some cells ‘unwanted’ for elimination by phagocytic macrophages via specific complement receptors. The complement protein, it should be noted, is extremely localized to immature synapses (75). Another study suggested that less active synapses are tagged with the C3 protein, after which they are pruned by microglia via the phagocytic signaling pathway (16).

Our study demonstrated that within 4 days, fear-induction led to significant prelimbic microglial activation, with associated alterations in cellular morphology and phagocytic marker expressions. A previous study reported that fear conditioning altered neuronal structural plasticity in adult hippocampal CA1 pyramidal neurons by enhancing dendritic ramifications and modulating synaptic density, which is closely associated with Semaphorin 4C/Plexin-B2 signaling. Upon studying neuronal morphology of CA1 neurons with Sholl analyses, a notable increase of the total length of both apical and basal dendrites were observed on day 2 after exposed to foot-shock (76). Additionally, our study exhibited another possibility that fear conditioning induced excessive synaptic pruning in the prelimbic through TREM2. Golgi-Cox staining in rats revealed that early-life stress led to a higher density of prominent spines compared with controls on postnatal days 15 and 50, and that this effect was more pronounced in females than males. While our study found mice showed deceased spine density after exposing to foot-shock. Rats exposed to early-life stress also displayed less co-localization with PSD95 and more pronounced freezing level during auditory fear memory testing (77). Another study similarly showed that early-life inflammation leads to adolescent depressive-like symptoms via mediating the phagocytic activity of microglia around ACC (78). Additionally, increased CSF1R on microglia increases phagocytosis of neuronal elements in the medial prefrontal cortex after chronic stress exposure (79). Herein, we extend previous work by showing that fear conditioning induced increased number of SYP+ and PSD95+puncta inside microglia or CD68, while inhibiting microglial phagocytosis via PLX5622 and minocycline rescued this trend. Moreover, restraining microglia reduced neuronal activity and mouse fear-related response. Thus, our findings reveal that altered prelimbic microglial phagocytosis contributes to spinal density and glutamatergic neuronal activity, eventually regulating the fear memory formation process.

Individuals with loss-of-function alleles at the CSF1R locus almost completely lose microglia, the M*ϕ*s of the brain, and have deficiencies in skeletal development and osteosclerosis. To our knowledge, there are no reported cases of patients with the homozygous loss-of-function mutation in CSF1 (80). DNAX-activating protein of 12kDa (DAP12) forms a molecular complex with TREM2, and the activation of DAP12/TREM-2 is involved in the process of differentiation of macrophages, DCs and microglial cells under inflammatory conditions (81). It has been reported that loss-of-function mutations in the DAP12 gene are responsible for presenile dementia and bone cysts. Moreover, it has been suggested in some cases that the mutation deletes four of the five exons of DAP12 (82). TREM2 is among the most important microglial surface receptors, the absence of which impairs microglia-mediated pruning in the developing brain and in some neurodegenerative disorders (83, 84). Homozygous loss-of-function mutations in TREM2 seem to be a vital risk factor for late-onset Alzheimer’s disease (85), and a significant association has been reported between TREM2 and Parkinson’s disease, amyotrophic lateral sclerosis, and frontotemporal dementia (86–88). Because of its control over microglial phagocytosis, TREM2 may play a protective role, though it may be involved differently in diseases like Alzheimer’s disease (89). In Aβ (1–42) lesioned rats, upregulated expression of TREM2 was observed in the hippocampus on post-lesion days 3, 7, 10, and 14 compared with the vehicle-injected group. Moreover, these data provide credible evidence of a positive correlation between the relative expression TREM2 mRNA and contextual freezing time. That group found that memory is enhanced with increased TREM2 mRNA level similar to our results that decreased freezing level occurred in Trem2 KO mice compared to WT mice (90). During Morris water maze test, APPswe/PS1dE9 mice displayed spatial learning and memory deficit, which were subsequently found improved behavioral performance as inserting of TREM2 vector into these mice (91, 92). Our study suggested that TREM2 strengthens the process of fear memory formation via the regulation of microglial pruning of superfluous synapses and facilitation the remaining synapses, which subsequently activated the glutamatergic neurons. Of note, synaptic pruning impairments detected in Trem2 KO mice were paralleled by fewer activated prelimbic microglia. Reduced SYP+ and PSD95+puncta inside microglia or CD68 were discovered after Trem2 deletion, as was increased dendritic spine density. Postmortem analyses of patients with PTSD have also shown reduced dendrites/spines, or increased immature spines, in the medial prefrontal cortex (93).

In conclusion, TREM2 in the prelimbic correlates with regulation of fear formation via modulating microglia-mediated excessive synaptic pruning and glutamatergic neuron activity. Inhibiting the glutamatergic neurons or activated microglia in the prelimbic mitigates fear memory formation. Increasing understanding of the tripartite interactions among TREM2, microglia, and synapses during fear conditioning may eventually allow us to reduce fear memory formation through TREM2 inhibition, and thus reduce synapse loss, toward developing effective cures for patients with fear-related psychiatric disorders.

## Data Availability

The original contributions presented in the study are included in the article/**Supplementary Materials**. Further inquiries can be directed to the corresponding authors.

## Supplementary material

Supplementary Figure 1
**(A)** Representative confocal images of Iba1 immunostaining (green) of microglia in the prelimbic of control, 1 Day, 4Day, 7Day after foot-shock exposure. Scale bars =50 μm. **(B)** Sholl analysis of microglial morphology of control and fear conditioning group; note the reduced arborization in 4 days after foot-shock (n=3 mice, 10 cells per mice. **P < 0.01, ***P < 0.001). **(C)** Representative confocal images of Iba1 immunostaining (green) of microglia in the infralimbic of control and fear conditioning group. Scale bars =50 μm. **(D)** Sholl analysis of microglial morphology of control and fear conditioning group (n=3 mice, 10 cells per mice. ***P < 0.001). **(E)** Representative confocal images of Iba1 immunostaining (green) of microglia in the hippocampus of control and fear conditioning group. Scale bars =50 μm. **(F)** Sholl analysis of microglial morphology of control and fear conditioning group (n=3 mice, 10 cells per mice).

Supplementary Figure 2
**(A)** Representative images of c-Fos (red) and CaMKII (green) in infralimbic of control and FC mice (scale bars = 100 μm [first row]; 20 μm [the other rows]). **(B)** Number of infralimbic CaMKII and c-Fos double-labeled neurons (n = 3 mice, 2 slices per mouse). **(C)** Representative images of prelimbic negative staining (scale bars = 100 μm [first row]; 20 μm [the other rows]).

Supplementary Figure 3
**(A)** Relative prelimbic proinflammatory cytokine mRNA expression (n = 6 mice, **P < 0.01, ***P < 0.001).

Supplementary Figure 4
**(A)** The percentage of freezing in mice with minocycline microinjection in the prelimbic during fear conditioning (n = 8 mice. *P < 0.05). **(B, C)** The percentage of freezing in mice with minocycline microinjection in the prelimbic during the contextual **(B)** and cued **(C)** fear memory test (n = 8 mice. *P < 0.05, **P < 0.01).

Supplementary Figure 5
**(A)** The percentage of freezing in mice with Trem2 siRNA microinjection in the prelimbic during fear conditioning (n = n = 8 mice. ***P < 0.001). **(B, C)** The percentage of freezing in mice with Trem2 siRNA microinjection in the prelimbic during the contextual **(B)** and cued **(C)** fear memory test (n = 8 mice. **P < 0.01, ***P < 0.001).

## References

[1] Bocchio M Nabavi S Capogna M. Synaptic plasticity, engrams, and network oscillations in amygdala circuits for storage and retrieval of emotional memories. Neuron. (2017) 94:731–43. doi: 10.1016/j.neuron.2017.03.022 28521127

[2] Careaga MBL Girardi CEN Suchecki D. Understanding posttraumatic stress disorder through fear conditioning, extinction and reconsolidation. Neurosci Biobehav Rev. (2016) 71:48–57. doi: 10.1016/j.neubiorev.2016.08.023 27590828

[3] Ilovich O Dines M Paul BK Barkai E Lamprecht R. Nck1 activity in lateral amygdala regulates long-term fear memory formation. Trans Psychiatry. (2022) 12:475. doi: 10.1038/s41398-022-02244-x PMC9653413 36371406

[4] Enomoto S Kato TA. Involvement of microglia in disturbed fear memory regulation: possible microglial contribution to the pathophysiology of posttraumatic stress disorder. Neurochem Int. (2021) 142:104921. doi: 10.1016/j.neuint.2020.104921 33232758

[5] Chen MB Jiang X Quake SR Sudhof TC. Persistent transcriptional programmes are associated with remote memory. Nature. (2020) 587:437–42. doi: 10.1038/s41586-020-2905-5 PMC9097329 33177708

[6] Cummings KA Bayshtok S Dong TN Kenny PJ Clem RL. Control of fear by discrete prefrontal gabaergic populations encoding valence-specific information. Neuron. (2022) 110:3036–52 e5. doi: 10.1016/j.neuron.2022.07.004 35944526 PMC10009874

[7] Cummings KA Clem RL. Prefrontal somatostatin interneurons encode fear memory. Nat Neurosci. (2020) 23:61–74. doi: 10.1038/s41593-019-0552-7 31844314 PMC6930333

[8] Fan X Song J Ma C Lv Y Wang F Ma L. Noradrenergic signaling mediates cortical early tagging and storage of remote memory. Nat Commun. (2022) 13:7623. doi: 10.1038/s41467-022-35342-x 36494350 PMC9734098

[9] Sung Y Kaang BK. The three musketeers in the medial prefrontal cortex: subregion-specific structural and functional plasticity underlying fear memory stages. Exp Neurobiol. (2022) 31:221–31. doi: 10.5607/en22012 PMC9471411 36050222

[10] Sun W Yang Y Chen X Cheng Y Li X An L. Light promotes neural correlates of fear memory via enhancing brain-derived neurotrophic factor (Bdnf) expression in the prelimbic cortex. ACS Chem Neurosci. (2021) 12:1802–10. doi: 10.1021/acschemneuro.1c00081 33961393

[11] Sierra-Mercado D Padilla-Coreano N Quirk GJ. Dissociable roles of prelimbic and infralimbic cortices, ventral hippocampus, and basolateral amygdala in the expression and extinction of conditioned fear. Neuropsychopharmacology. (2011) 36:529–38. doi: 10.1038/npp.2010.184 PMC3005957 20962768

[12] Giustino TF Maren S. The role of the medial prefrontal cortex in the conditioning and extinction of fear. Front Behav Neurosci. (2015) 9:298. doi: 10.3389/fnbeh.2015.00298 26617500 PMC4637424

[13] Prinz M Erny D Hagemeyer N. Ontogeny and homeostasis of Cns myeloid cells. Nat Immunol. (2017) 18:385–92. doi: 10.1038/ni.3703 28323268

[14] Salter MW Stevens B. Microglia emerge as central players in brain disease. Nat Med. (2017) 23:1018–27. doi: 10.1038/nm.4397 28886007

[15] Crotti A Ransohoff RM. Microglial physiology and pathophysiology: insights from genome-wide transcriptional profiling. Immunity. (2016) 44:505–15. doi: 10.1016/j.immuni.2016.02.013 26982357

[16] Schafer DP Lehrman EK Kautzman AG Koyama R Mardinly AR Yamasaki R. Microglia sculpt postnatal neural circuits in an activity and complement-dependent manner. Neuron. (2012) 74:691–705. doi: 10.1016/j.neuron.2012.03.026 22632727 PMC3528177

[17] Stevens B Allen NJ Vazquez LE Howell GR Christopherson KS Nouri N. The classical complement cascade mediates Cns synapse elimination. Cell. (2007) 131:1164–78. doi: 10.1016/j.cell.2007.10.036 18083105

[18] Wilton DK Dissing-Olesen L Stevens B. Neuron-glia signaling in synapse elimination. Annu Rev Neurosci. (2019) 42:107–27. doi: 10.1146/annurev-neuro-070918-050306 31283900

[19] Han X Xu T Ding C Wang D Yao G Chen H. Neuronal Nr4a1 deficiency drives complement-coordinated synaptic stripping by microglia in a mouse model of lupus. Signal Transduct Target Ther. (2022) 7:328. doi: 10.1038/s41392-021-00867-y 35177587 PMC8854434

[20] Ding X Wang J Huang M Chen Z Liu J Zhang Q. Loss of microglial sirpalpha promotes synaptic pruning in preclinical models of neurodegeneration. Nat Commun. (2021) 12:2030. doi: 10.1038/s41467-021-22301-1 33795678 PMC8016980

[21] Aono H Choudhury ME Higaki H Miyanishi K Kigami Y Fujita K. Microglia may compensate for dopaminergic neuron loss in experimental parkinsonism through selective elimination of glutamatergic synapses from the subthalamic nucleus. Glia. (2017) 65:1833–47. doi: 10.1002/glia.23199 28836295

[22] Cui X Zhou S Xia G Chen J Jiang L Huang J. A multispecies probiotic accelerates fear extinction and inhibits relapse in mice: role of microglia. Neuropharmacology. (2021) 193:108613. doi: 10.1016/j.neuropharm.2021.108613 34022177

[23] Smith KL Kassem MS Clarke DJ Kuligowski MP Bedoya-Perez MA Todd SM. Microglial cell hyper-ramification and neuronal dendritic spine loss in the hippocampus and medial prefrontal cortex in a mouse model of Ptsd. Brain Behav Immun. (2019) 80:889–99. doi: 10.1016/j.bbi.2019.05.042 31158497

[24] Sharif O Knapp S. From expression to signaling: roles of Trem-1 and Trem-2 in innate immunity and bacterial infection. Immunobiology. (2008) 213:701–13. doi: 10.1016/j.imbio.2008.07.008 18926286

[25] Colonna M. Trems in the immune system and beyond. Nat Rev Immunol. (2003) 3:445–53. doi: 10.1038/nri1106 12776204

[26] Ford JW McVicar DW. Trem and Trem-like receptors in inflammation and disease. Curr Opin Immunol. (2009) 21:38–46. doi: 10.1016/j.coi.2009.01.009 19230638 PMC2723941

[27] Ulland TK Song WM Huang SC Ulrich JD Sergushichev A Beatty WL. Trem2 maintains microglial metabolic fitness in Alzheimer’s disease. Cell. (2017) 170:649–63 e13. doi: 10.1016/j.cell.2017.07.023 28802038 PMC5573224

[28] Konishi H Kiyama H. Microglial Trem2/Dap12 signaling: A double-edged sword in neural diseases. Front Cell Neurosci. (2018) 12:206. doi: 10.3389/fncel.2018.00206 30127720 PMC6087757

[29] Derecki NC Cronk JC Lu Z Xu E Abbott SB Guyenet PG. Wild-type microglia arrest pathology in a mouse model of Rett syndrome. Nature. (2012) 484:105–9. doi: 10.1038/nature10907 PMC3321067 22425995

[30] He D Xu H Zhang H Tang R Lan Y Xing R. Disruption of the Il-33-St2-Akt signaling axis impairs neurodevelopment by inhibiting microglial metabolic adaptation and phagocytic function. Immunity. (2022) 55:159–73 e9. doi: 10.1016/j.immuni.2021.12.001 34982959 PMC9074730

[31] Dong Y Li S Lu Y Li X Liao Y Peng Z. Stress-induced Nlrp3 inflammasome activation negatively regulates fear memory in mice. J Neuroinflamm. (2020) 17:205. doi: 10.1186/s12974-020-01842-0 PMC7341659 32635937

[32] Zhao N Liu C-C Qiao W Bu G. Apolipoprotein E, receptors, and modulation of Alzheimer’s disease. Biol Psychiatry. (2018) 83:347–57. doi: 10.1016/j.biopsych.2017.03.003 PMC5599322 28434655

[33] Shirotani K Hori Y Yoshizaki R Higuchi E Colonna M Saito T. Aminophospholipids are signal-transducing Trem2 ligands on apoptotic cells. Sci Rep. (2019) 9:7508. doi: 10.1038/s41598-019-43535-6 31101881 PMC6525155

[34] Zhang G Ásgeirsdóttir HN Cohen SJ Munchow AH Barrera MP Stackman RW. Stimulation of serotonin 2a receptors facilitates consolidation and extinction of fear memory in C57bl/6j mice. Neuropharmacology. (2013) 64:403–13. doi: 10.1016/j.neuropharm.2012.06.007 PMC3477617 22722027

[35] Peng X Chen P Zhang Y Wu K Ji N Gao J. Mpp2 interacts with Sk2 to rescue the excitability of glutamatergic neurons in the Bla and facilitate the extinction of conditioned fear in mice. CNS Neurosci Ther. (2023) 30:e14362. doi: 10.1111/cns.14362 37469037 PMC10805397

[36] Wu K Y-y L Shao S Song W Chen X-H Dong Y-T. The microglial innate immune receptors Trem-1 and Trem-2 in the anterior cingulate cortex (Acc) drive visceral hypersensitivity and depressive-like behaviors following Dss-induced colitis. Brain Behav Immun. (2023) 112:96–117. doi: 10.1016/j.bbi.2023.06.003 37286175

[37] Benitez DP Jiang S Wood J Wang R Hall CM Peerboom C. Knock-in models related to Alzheimer’s disease: synaptic transmission, plaques and the role of microglia. Mol Neurodegener. (2021) 16:47. doi: 10.1186/s13024-021-00457-0 34266459 PMC8281661

[38] Huang Y Xu Z Xiong S Sun F Qin G Hu G. Repopulated microglia are solely derived from the proliferation of residual microglia after acute depletion. Nat Neurosci. (2018) 21:530–40. doi: 10.1038/s41593-018-0090-8 29472620

[39] Li S Liao Y Dong Y Li X Li J Cheng Y. Microglial deletion and inhibition alleviate behavior of post-traumatic stress disorder in mice. J Neuroinflamm. (2021) 18:7. doi: 10.1186/s12974-020-02069-9 PMC7786489 33402212

[40] Wang J Chen HS Li HH Wang HJ Zou RS Lu XJ. Microglia-dependent excessive synaptic pruning leads to cortical underconnectivity and behavioral abnormality following chronic social defeat stress in mice. Brain Behav Immun. (2023) 109:23–36. doi: 10.1016/j.bbi.2022.12.019 36581303

[41] Socodato R Portugal CC Canedo T Rodrigues A Almeida TO Henriques JF. Microglia dysfunction caused by the loss of rhoa disrupts neuronal physiology and leads to neurodegeneration. Cell Rep. (2020) 31:107796. doi: 10.1016/j.celrep.2020.107796 32579923

[42] Hickman SE Kingery ND Ohsumi TK Borowsky ML Wang L-C Means TK. The microglial sensome revealed by direct Rna sequencing. Nat Neurosci. (2013) 16:1896–905. doi: 10.1038/nn.3554 PMC3840123 24162652

[43] Zhong L Sheng X Wang W Li Y Zhuo R Wang K. Trem2 receptor protects against complement-mediated synaptic loss by binding to complement C1q during neurodegeneration. Immunity. (2023) 56:1794–808.e8. doi: 10.1016/j.immuni.2023.06.016 37442133

[44] Jay TR von Saucken VE Muñoz B Codocedo JF Atwood BK Lamb BT. Trem2 is required for microglial instruction of astrocytic synaptic engulfment in neurodevelopment. Glia. (2019) 67:1873–92. doi: 10.1002/glia.23664 31265185

[45] Nugent AA Lin K van Lengerich B Lianoglou S Przybyla L Davis SS. Trem2 regulates microglial cholesterol metabolism upon chronic phagocytic challenge. Neuron. (2020) 105:837–54.e9. doi: 10.1016/j.neuron.2019.12.007 31902528

[46] Perrine SA Eagle AL George SA Mulo K Kohler RJ Gerard J. Severe, multimodal stress exposure induces Ptsd-like characteristics in a mouse model of single prolonged stress. Behav Brain Res. (2016) 303:228–37. doi: 10.1016/j.bbr.2016.01.056 26821287

[47] Xue B Xue J Yu Y Wei S-G Beltz TG Felder RB. Predator scent-induced sensitization of hypertension and anxiety-like behaviors. Cell Mol Neurobiol. (2020) 42:1141–52. doi: 10.1007/s10571-020-01005-y PMC8126575 33201417

[48] Jelenik T Dille M Müller-Lühlhoff S Kabra DG Zhou Z Binsch C. Fgf21 regulates insulin sensitivity following long-term chronic stress. Mol Metab. (2018) 16:126–38. doi: 10.1016/j.molmet.2018.06.012 PMC6158095 29980484

[49] Bali A Jaggi AS. Electric foot shock stress: A useful tool in neuropsychiatric studies. Rev Neurosci. (2015) 26:655–77. doi: 10.1515/revneuro-2015-0015 26167978

[50] Liao Z Tao Y Guo X Cheng D Wang F Liu X. Fear conditioning downregulates Rac1 activity in the basolateral amygdala astrocytes to facilitate the formation of fear memory. Front Mol Neurosci. (2017) 10:396. doi: 10.3389/fnmol.2017.00396 29230165 PMC5712045

[51] You Y Chen Z Hu W-W. The role of microglia heterogeneity in synaptic plasticity and brain disorders: will sequencing shed light on the discovery of new therapeutic targets? Pharmacol Ther. (2024) 255:108606. doi: 10.1016/j.pharmthera.2024.108606 38346477

[52] Keren-Shaul H Spinrad A Weiner A Matcovitch-Natan O Dvir-Szternfeld R Ulland TK. A unique microglia type associated with restricting development of Alzheimer’s disease. Cell. (2017) 169:1276–90 e17. doi: 10.1016/j.cell.2017.05.018 28602351

[53] Krasemann S Madore C Cialic R Baufeld C Calcagno N El Fatimy R. The Trem2-apoe pathway drives the transcriptional phenotype of dysfunctional microglia in neurodegenerative diseases. Immunity. (2017) 47:566–81 e9. doi: 10.1016/j.immuni.2017.08.008 28930663 PMC5719893

[54] Sala Frigerio C Wolfs L Fattorelli N Thrupp N Voytyuk I Schmidt I. The major risk factors for Alzheimer’s disease: age, sex, and genes modulate the microglia response to Aβ Plaques. Cell Rep. (2019) 27:1293–306 e6. doi: 10.1016/j.celrep.2019.03.099 31018141 PMC7340153

[55] Absinta M Maric D Gharagozloo M Garton T Smith MD Jin J. A lymphocyte-microglia-astrocyte axis in chronic active multiple sclerosis. Nature. (2021) 597:709–14. doi: 10.1038/s41586-021-03892-7 PMC8719282 34497421

[56] Marschallinger J Iram T Zardeneta M Lee SE Lehallier B Haney MS. Lipid-droplet-accumulating microglia represent a dysfunctional and proinflammatory state in the aging brain. Nat Neurosci. (2020) 23:194–208. doi: 10.1038/s41593-019-0566-1 31959936 PMC7595134

[57] De Andrade Costa A Chatterjee J Cobb O Sanapala S Scheaffer S Guo X. Rna sequence analysis reveals Itgal/Cd11a as a stromal regulator of murine low-grade glioma growth. Neuro Oncol. (2022) 24:14–26. doi: 10.1093/neuonc/noab130 34043012 PMC8730775

[58] Paolicelli RC Sierra A Stevens B Tremblay M-E Aguzzi A Ajami B. Microglia states and nomenclature: A field at its crossroads. Neuron. (2022) 110:3458–83. doi: 10.1016/j.neuron.2022.10.020 PMC9999291 36327895

[59] Sobue A Komine O Hara Y Endo F Mizoguchi H Watanabe S. Microglial gene signature reveals loss of homeostatic microglia associated with neurodegeneration of Alzheimer’s disease. Acta Neuropathol Commun. (2021) 9:1. doi: 10.1186/s40478-020-01099-x 33402227 PMC7786928

[60] Chiu IM Morimoto ET Goodarzi H Liao JT O’Keeffe S Phatnani HP. A neurodegeneration-specific gene-expression signature of acutely isolated microglia from an amyotrophic lateral sclerosis mouse model. Cell Rep. (2013) 4:385–401. doi: 10.1016/j.celrep.2013.06.018 23850290 PMC4272581

[61] Safaiyan S Besson-Girard S Kaya T Cantuti-Castelvetri L Liu L Ji H. White matter aging drives microglial diversity. Neuron. (2021) 109:1100–17 e10. doi: 10.1016/j.neuron.2021.01.027 33606969

[62] Mendoza C Barreto GE Ávila-Rodriguez M Echeverria V. Role of neuroinflammation and sex hormones in war-related Ptsd. Mol Cell Endocrinol. (2016) 434:266–77. doi: 10.1016/j.mce.2016.05.016 27216917

[63] Sadeghi MA Hemmati S Yousefi-Manesh H Foroutani L Nassireslami E Yousefi Zoshk M. Cilostazol pretreatment prevents Ptsd-related anxiety behavior through reduction of hippocampal neuroinflammation. Naunyn Schmiedebergs Arch Pharmacol. (2023) 397:133–44. doi: 10.1007/s00210-023-02578-3 37382600

[64] Shanazz K Nalloor R Lucas R Vazdarjanova A. Neuroinflammation is a susceptibility factor in developing a Ptsd-like phenotype. Front Behav Neurosci. (2023) 17:1112837. doi: 10.3389/fnbeh.2023.1112837 37064304 PMC10090279

[65] Schramm E Waisman A. Microglia as central protagonists in the chronic stress response. Neurol Neuroimmunol Neuroinflamm. (2022) 9:e200023. doi: 10.1212/NXI.0000000000200023 36357946 PMC9453699

[66] Dantzer R. From stress sensitization to microglial priming and vice versa: A new era of research in biological psychiatry. Biol Psychiatry. (2019) 85:619–20. doi: 10.1016/j.biopsych.2019.02.002 PMC6544015 30922465

[67] Wilson CB McLaughlin LD Nair A Ebenezer PJ Dange R Francis J. Inflammation and oxidative stress are elevated in the brain, blood, and adrenal glands during the progression of post-traumatic stress disorder in a predator exposure animal model. PloS One. (2013) 8:e76146. doi: 10.1371/journal.pone.0076146 24130763 PMC3794007

[68] Deslauriers J Powell S Risbrough VB. Immune signaling mechanisms of Ptsd risk and symptom development: insights from animal models. Curr Opin Behav Sci. (2017) 14:123–32. doi: 10.1016/j.cobeha.2017.01.005 PMC5525319 28758144

[69] Morrison FG Miller MW Wolf EJ Logue MW Maniates H Kwasnik D. Reduced interleukin 1a gene expression in the dorsolateral prefrontal cortex of individuals with Ptsd and depression. Neurosci Lett. (2019) 692:204–9. doi: 10.1016/j.neulet.2018.10.027 PMC6351202 30366016

[70] Bhatt S Hillmer AT Girgenti MJ Rusowicz A Kapinos M Nabulsi N. Ptsd is associated with neuroimmune suppression: evidence from pet imaging and postmortem transcriptomic studies. Nat Commun. (2020) 11:2360. doi: 10.1038/s41467-020-15930-5 32398677 PMC7217830

[71] Paolicelli RC Bolasco G Pagani F Maggi L Scianni M Panzanelli P. Synaptic pruning by microglia is necessary for normal brain development. Science. (2011) 333:1456–8. doi: 10.1126/science.1202529 21778362

[72] Kettenmann H Kirchhoff F Verkhratsky A. Microglia: new roles for the synaptic stripper. Neuron. (2013) 77:10–8. doi: 10.1016/j.neuron.2012.12.023 23312512

[73] Riccomagno MM Kolodkin AL. Sculpting neural circuits by axon and dendrite pruning. Annu Rev Cell Dev Biol. (2015) 31:779–805. doi: 10.1146/annurev-cellbio-100913-013038 26436703 PMC4668927

[74] Milinkeviciute G Henningfield CM Muniak MA Chokr SM Green KN Cramer KS. Microglia regulate pruning of specialized synapses in the auditory brainstem. Front Neural Circuits. (2019) 13:55. doi: 10.3389/fncir.2019.00055 31555101 PMC6722190

[75] Koyama R Ikegaya Y. Microglia in the pathogenesis of autism spectrum disorders. Neurosci Res. (2015) 100:1–5. doi: 10.1016/j.neures.2015.06.005 26116891

[76] Simonetti M Paldy E Njoo C Bali KK Worzfeld T Pitzer C. The impact of semaphorin 4c/plexin-B2 signaling on fear memory via remodeling of neuronal and synaptic morphology. Mol Psychiatry. (2019) 26:1376–98. doi: 10.1038/s41380-019-0491-4 PMC7985029 31444474

[77] Zetter MA Hernández VS Roque A Hernández-Pérez OR Gómora MJ Ruiz-Velasco S. Microglial synaptic pruning on axon initial segment spines of dentate granule cells: sexually dimorphic effects of early-life stress and consequences for adult fear response. J Neuroendocrinol. (2021) 33:e12969. doi: 10.1111/jne.12969 33890333 PMC13186562

[78] Cao P Chen C Liu A Shan Q Zhu X Jia C. Early-life inflammation promotes depressive symptoms in adolescence via microglial engulfment of dendritic spines. Neuron. (2021) 109:2573–89.e9. doi: 10.1016/j.neuron.2021.06.012 34233151

[79] Wohleb ES Terwilliger R Duman CH Duman RS. Stress-induced neuronal colony stimulating factor 1 provokes microglia-mediated neuronal remodeling and depressive-like behavior. Biol Psychiatry. (2018) 83:38–49. doi: 10.1016/j.biopsych.2017.05.026 28697890 PMC6506225

[80] Hume DA Caruso M Ferrari-Cestari M Summers KM Pridans C Irvine KM. Phenotypic impacts of Csf1r deficiencies in humans and model organisms. J Leukocyte Biol. (2020) 107:205–19. doi: 10.1002/jlb.Mr0519-143r 31330095

[81] Colonna M. Dap12 signaling: from immune cells to bone modeling and brain myelination. J Clin Invest. (2003) 111:313–4. doi: 10.1172/JCI17745 PMC151875 12569153

[82] Paloneva J Kestilä M Wu J Salminen A Böhling T Ruotsalainen V. Loss-of-function mutations in Tyrobp (Dap12) result in a presenile dementia with bone cysts. Nat Genet. (2000) 25:357–61. doi: 10.1038/77153 10888890

[83] Filipello F Morini R Corradini I Zerbi V Canzi A Michalski B. The microglial innate immune receptor Trem2 is required for synapse elimination and normal brain connectivity. Immunity. (2018) 48:979–91.e8. doi: 10.1016/j.immuni.2018.04.016 29752066

[84] Dayananda KK Ahmed S Wang D Polis B Islam R Kaffman A. Early life stress impairs synaptic pruning in the developing hippocampus. Brain Behav Immun. (2023) 107:16–31. doi: 10.1016/j.bbi.2022.09.014 36174883 PMC10497209

[85] Guerreiro R Wojtas A Bras J Carrasquillo M Rogaeva E Majounie E. Trem2 variants in Alzheimer’s disease. N Engl J Med. (2013) 368:117–27. doi: 10.1056/NEJMoa1211851 PMC3631573 23150934

[86] Cady J Koval ED Benitez BA Zaidman C Jockel-Balsarotti J Allred P. Trem2 variant P.R47h as a risk factor for sporadic amyotrophic lateral sclerosis. JAMA Neurol. (2014) 71:449–53. doi: 10.1001/jamaneurol.2013.6237 PMC4087113 24535663

[87] Kober DL Alexander-Brett JM Karch CM Cruchaga C Colonna M Holtzman MJ. Neurodegenerative disease mutations in trem2 reveal a functional surface and distinct loss-of-function mechanisms. Elife. (2016) 5:e20391. doi: 10.7554/eLife.20391 27995897 PMC5173322

[88] Rayaprolu S Mullen B Baker M Lynch T Finger E Seeley WW. Trem2 in neurodegeneration: evidence for association of the P.R47h variant with frontotemporal dementia and Parkinson’s disease. Mol Neurodegener. (2013) 8:19. doi: 10.1186/1750-1326-8-19 23800361 PMC3691612

[89] Yu CJ Wang M Li RY Wei T Yang HC Yin YS. Trem2 and microglia contribute to the synaptic plasticity: from physiology to pathology. Mol Neurobiol. (2023) 60:512–23. doi: 10.1007/s12035-022-03100-1 36318443

[90] Shallie OF Mabandla MV. Amyloid-beta (1-42) lesion of Ca1 rat dorsal hippocampus reduces contextual fear memory and increases expression of microglial genes regulating neuroinflammation. Behav Brain Res. (2020) 393:112795. doi: 10.1016/j.bbr.2020.112795 32619564

[91] Jiang T Tan L Zhu X-C Zhang Q-Q Cao L Tan M-S. Upregulation of Trem2 ameliorates neuropathology and rescues spatial cognitive impairment in a transgenic mouse model of Alzheimer’s disease. Neuropsychopharmacology. (2014) 39:2949–62. doi: 10.1038/npp.2014.164 PMC4229581 25047746

[92] Jiang T Zhang Y-D Chen Q Gao Q Zhu X-C Zhou J-S. Trem2 modifies microglial phenotype and provides neuroprotection in P301s tau transgenic mice. Neuropharmacology. (2016) 105:196–206. doi: 10.1016/j.neuropharm.2016.01.028 26802771

[93] Chen X Jiang Y Wang J Liu Y Xiao M Song C. Synapse impairment associated with enhanced apoptosis in post-traumatic stress disorder. Synapse. (2019) 74:e22134. doi: 10.1002/syn.22134 31562782

