# Supplementary figures and images for: The microglial innate immune receptor TREM2 participates in fear memory formation through excessive prelimbic cortical synaptic pruning
Source: Front Immunol. 2024 Oct 31;15:1412699. doi: 10.3389/fimmu.2024.1412699 (PMC11560470; doi:10.3389/fimmu.2024.1412699)

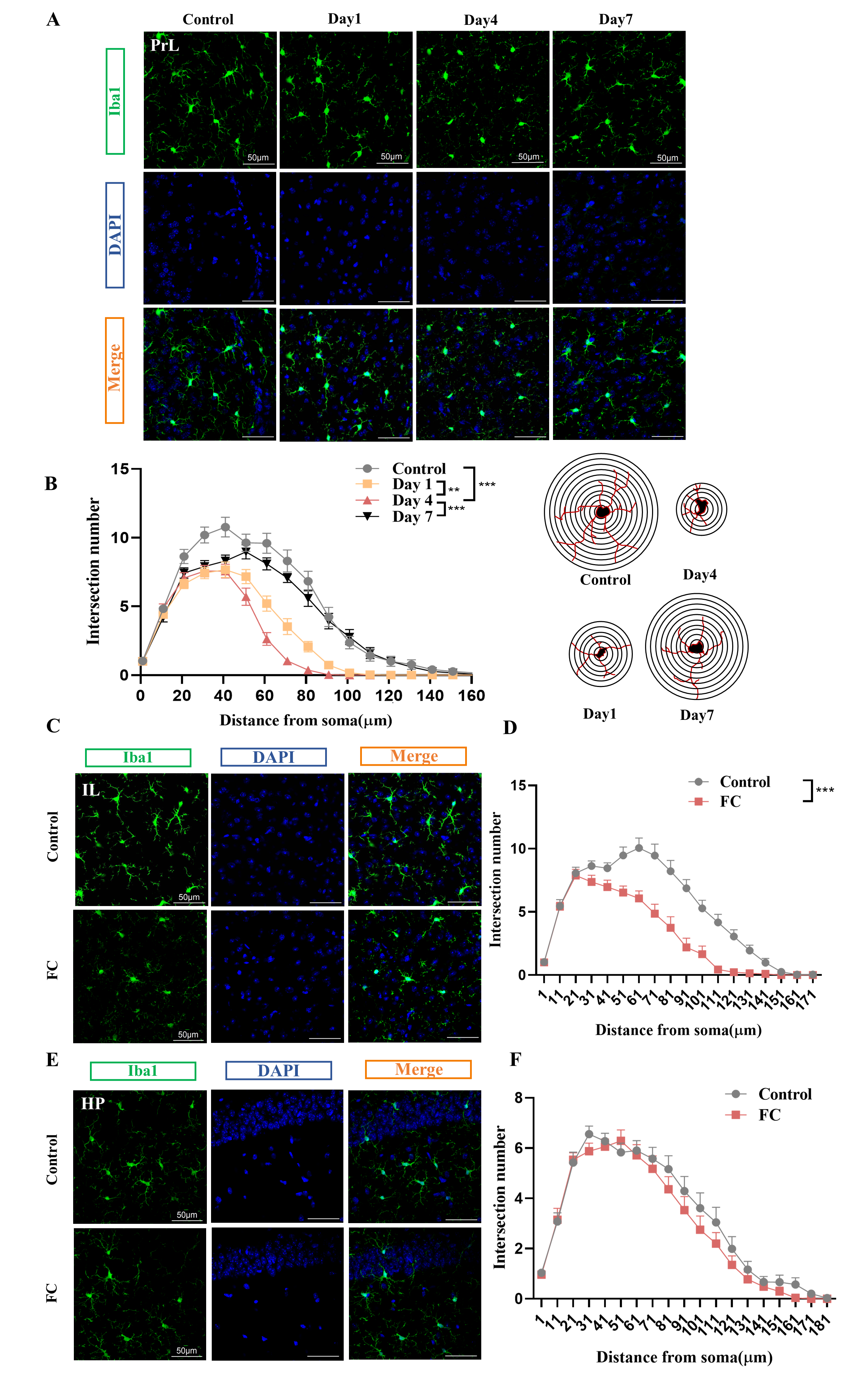

Supplement: Supplementary Figure 1 — (A) Representative confocal images of Iba1 immunostaining (green) of microglia in the prelimbic of control, 1 Day, 4Day, 7Day after foot-shock exposure. Scale bars =50 μm. (B) Sholl analysis of microglial morphology of control and fear conditioning group; note the reduced arborization in 4 days after foot-shock (n=3 mice, 10 cells per mice. **P < 0.01, ***P < 0.001). (C) Representative confocal images of Iba1 immunostaining (green) of microglia in the infralimbic of control and fear conditioning group. Scale bars =50 μm. (D) Sholl analysis of microglial morphology of control and fear conditioning group (n=3 mice, 10 cells per mice. ***P < 0.001). (E) Representative confocal images of Iba1 immunostaining (green) of microglia in the hippocampus of control and fear conditioning group. Scale bars =50 μm. (F) Sholl analysis of microglial morphology of control and fear conditioning group (n=3 mice, 10 cells per mice). [file Image1.tif]

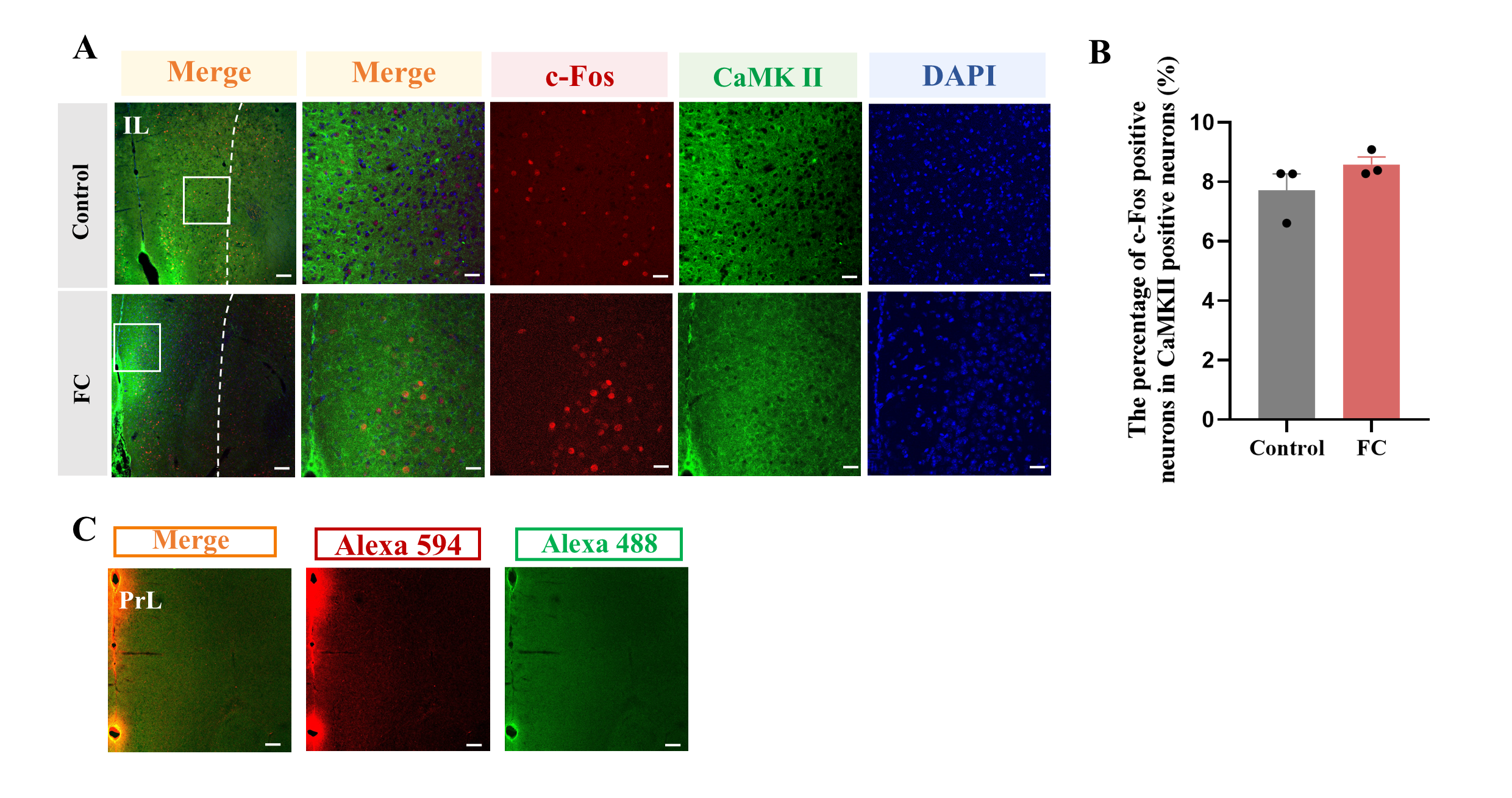

Supplement: Supplementary Figure 2 — (A) Representative images of c-Fos (red) and CaMKII (green) in infralimbic of control and FC mice (scale bars = 100 μm [first row]; 20 μm [the other rows]). (B) Number of infralimbic CaMKII and c-Fos double-labeled neurons (n = 3 mice, 2 slices per mouse). (C) Representative images of prelimbic negative staining (scale bars = 100 μm [first row]; 20 μm [the other rows]). [file Image2.tif]

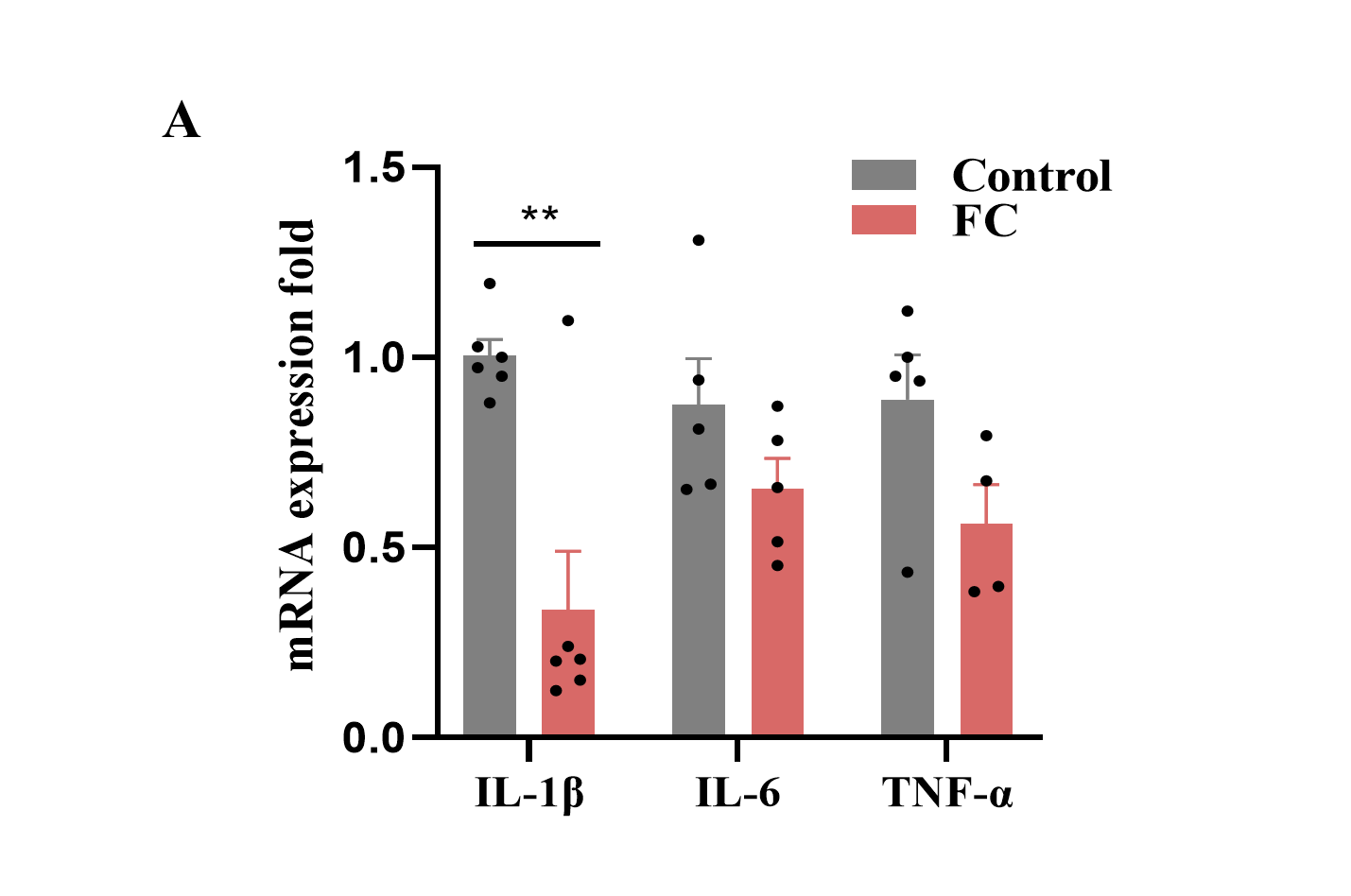

Supplement: Supplementary Figure 3 — (A) Relative prelimbic proinflammatory cytokine mRNA expression (n = 6 mice, **P < 0.01, ***P < 0.001). [file Image3.tif]

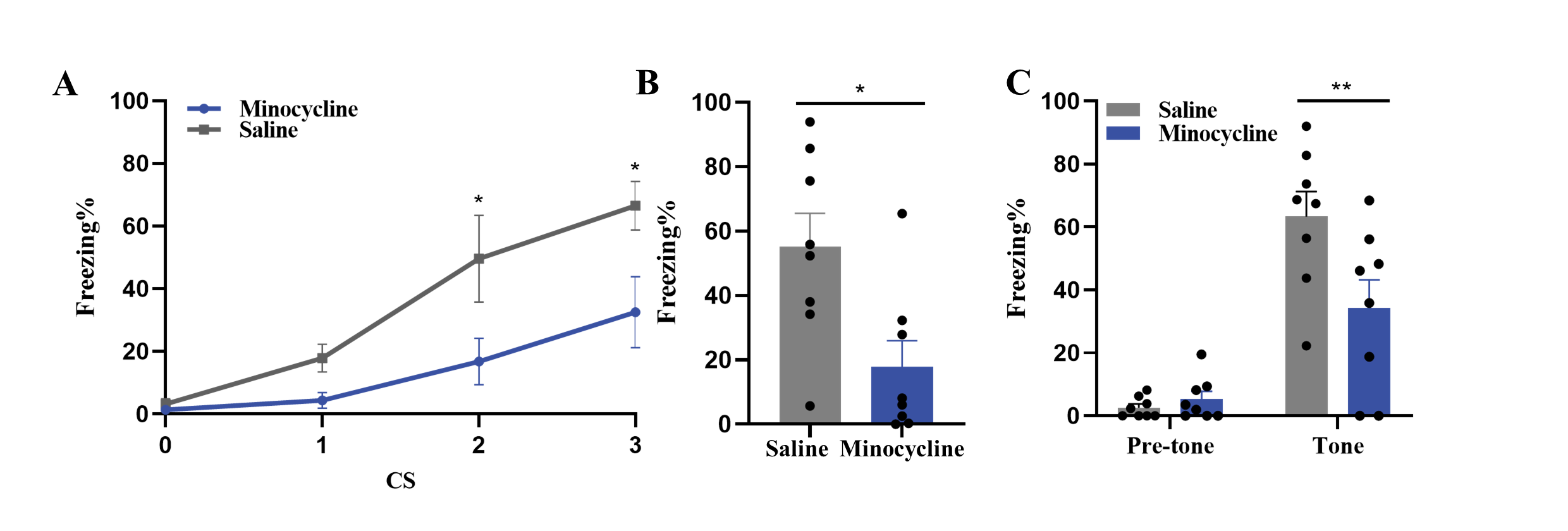

Supplement: Supplementary Figure 4 — (A) The percentage of freezing in mice with minocycline microinjection in the prelimbic during fear conditioning (n = 8 mice. *P < 0.05). (B, C) The percentage of freezing in mice with minocycline microinjection in the prelimbic during the contextual (B) and cued (C) fear memory test (n = 8 mice. *P < 0.05, **P < 0.01). [file Image4.tif]

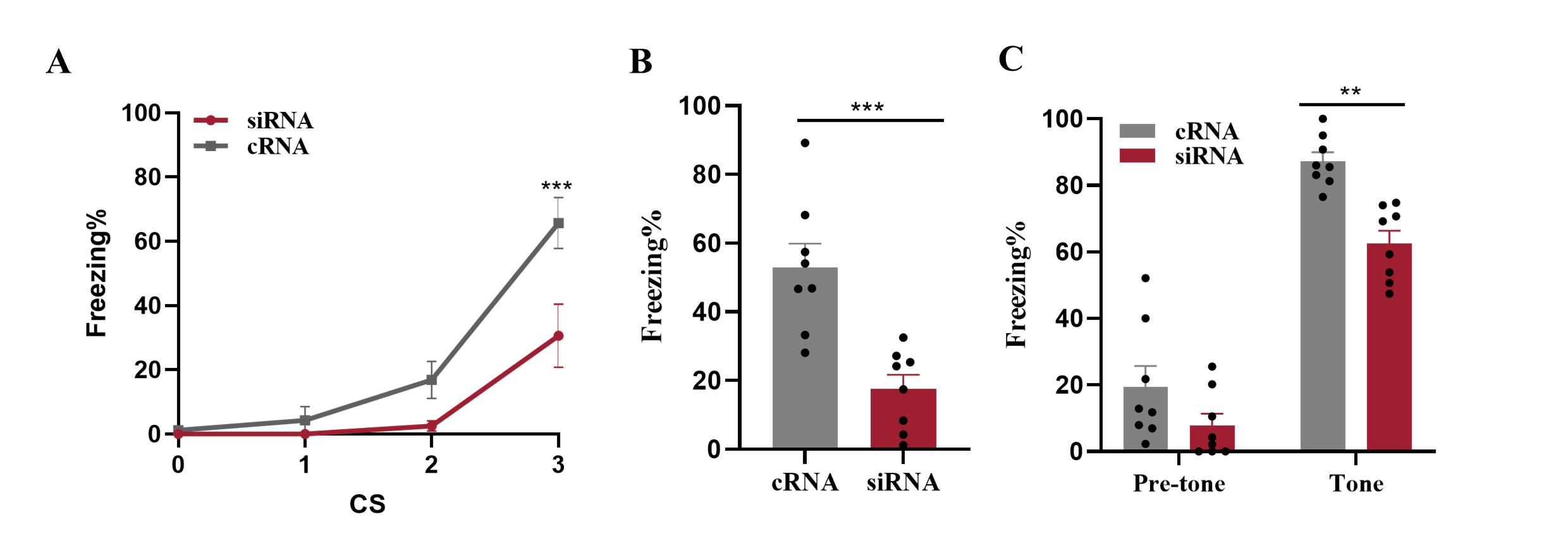

Supplement: Supplementary Figure 5 — (A) The percentage of freezing in mice with Trem2 siRNA microinjection in the prelimbic during fear conditioning (n = n = 8 mice. ***P < 0.001). (B, C) The percentage of freezing in mice with Trem2 siRNA microinjection in the prelimbic during the contextual (B) and cued (C) fear memory test (n = 8 mice. **P < 0.01, ***P < 0.001). [file Image5.tif]
